# Neural Patterns of the Implicit Association Test

**DOI:** 10.3389/fnhum.2015.00605

**Published:** 2015-11-24

**Authors:** Graham F. Healy, Lorraine Boran, Alan F. Smeaton

**Affiliations:** ^1^Insight Centre for Data Analytics, School of Computing, Dublin City UniversityDublin, Ireland; ^2^School of Nursing and Human Sciences, Dublin City UniversityDublin, Ireland

**Keywords:** event-related potentials, EEG, implicit association test, LORETA, brain regions, inhibition, word association, N200

## Abstract

The Implicit Association Test (IAT) is a reaction time based categorization task that measures the differential associative strength between bipolar targets and evaluative attribute concepts as an approach to indexing implicit beliefs or biases. An open question exists as to what exactly the IAT measures, and here EEG (Electroencephalography) has been used to investigate the time course of ERPs (Event-related Potential) indices and implicated brain regions in the IAT. IAT-EEG research identifies a number of early (250–450 ms) negative ERPs indexing early-(pre-response) processing stages of the IAT. ERP activity in this time range is known to index processes related to cognitive control and semantic processing. A central focus of these efforts has been to use IAT-ERPs to delineate the implicit and explicit factors contributing to measured IAT effects. Increasing evidence indicates that cognitive control (and related top-down modulation of attention/perceptual processing) may be components in the effective measurement of IAT effects, as factors such as physical setting or task instruction can change an IAT measurement. In this study we further implicate the role of proactive cognitive control and top-down modulation of attention/perceptual processing in the IAT-EEG. We find statistically significant relationships between D-score (a reaction-time based measure of the IAT-effect) and early ERP-time windows, indicating where more rapid word categorizations driving the IAT effect are present, they are at least partly explainable by neural activity not significantly correlated with the IAT measurement itself. Using LORETA, we identify a number of brain regions driving these ERP-IAT relationships notably involving left-temporal, insular, cingulate, medial frontal and parietal cortex in time regions corresponding to the N2- and P3-related activity. The identified brain regions involved with reduced reaction times on congruent blocks coincide with those of previous studies.

## 1. Introduction

The implicit-association test (IAT) is a measure of implicit bias based on the principle that if a congruent association between two concepts (e.g., target and stereotypical attribute) is readily accepted as accurate by a decision maker (e.g., disease → negative), then reaction time (RT) to categorizing such associations as equivalent is very rapid. In contrast, if an incongruent association between two concepts (e.g., target and counter-stereotypical attribute) is not readily accepted as accurate (e.g., disease → positive), then RT is comparatively slower due to inhibitory processes required to override an automatic tendency to associate congruent concepts. Response bias toward concept-pairings (e.g., fast responding to congruent; slow responding to incongruent) is not only influenced by knowledge of concrete characteristics (e.g., perceptual, functional) of bipolar concepts, but also by how we encode emotional valence in these concept associations though this is not always apparent in explicit self-report attitude measures (Greenwald and Banaji, 1995).

The IAT effect or measure of implicit bias is based on the standardized difference (D) between the mean RT to congruent and to incongruent pairings. A positive D-score indicates that individuals are either slow to respond to incongruent pairings, fast to respond to congruent pairings or a combination of both (Forbes et al., 2012). A decision maker's D-score can be used to measure a range of implicit beliefs reflecting social norms (Greenwald et al., 1998; Fazio and Olson, 2007), and these measures have proven effective in predicting later decision-making (Glasman and Albarracin, 2006).

Opponents of the IAT argue that issues like the low degree to which implicit IAT measures fail to corroborate explicit measures such as questionnaires, warrants strong consideration of what exactly the IAT is measuring (De Houwer et al., 2009). This line of evidence has been used to establish that the IAT reflects automatic beliefs through activation of stereotyped associations which are often dissociated from self-reported explicit beliefs (Greenwald et al., 1998), especially for socially sensitive topics due to factors such as social desirability (Hofmann et al., 2005). Despite reported dissociation between implicit and explicit beliefs, IAT measures show moderate correlation with explicit measures (Hofmann et al., 2005) and are known to be sensitive to a number of external influences (Boysen et al., 2006). Such studies ultimately indicate that the IAT captures meaningful information but its use must be considered with care.

An implicit measure of personal connectedness to nature based on latency to bipolar mappings of targets (“Me,” “Other”) and attributes (“Nature,” “Built”) is known as the n-IAT. Mean RT to congruent (e.g., “Nature-me”/“Built-Other”) and incongruent (e.g., “Built-Me”/“Nature-Other”) mappings are associated with emotional concern (e.g., anxiety) about the environment (Schultz et al., 2004). Bruni and Schultz (2010) reported strong associations in the n-IAT with natural relative to built environments among a sample of environmentalists. Despite observing similar high scores on self-reported measures of concern for the environment, significant correlations with explicit measures were restricted to a participant pool of college students and not environmental activists or children whom had higher n-IAT scores. Bruni et al. (2012) show the n-IAT is robust to framing effects and valence of the stimuli. In the n-IAT there are 4 categories of words used (“Me,” “Other,” “Nature,” “Built”). In compatible (congruent) trial blocks a participant is instructed to indicate by button press to which of the two category pairings (“Nature-Me” or “Built-Other”) the stimulus (word) belongs. In the incompatible (incongruent) trial blocks these category pairing are “Built-Me” or “Nature-Other.” Task switching is understood to exist in both of these block types as participants must switch between classifying stimuli as attributes (“Nature,” “Built”) or self-referential target categories (“Me,” “Other”).

The extent to which the IAT effect is caused by involuntary processes independent of the goal to inhibit pre-potent IAT responding remains unclear (De Houwer et al., 2009). Although a great deal of research has looked at faking IATs by manipulating response times (Verschuere et al., 2009), there has also been some success in faking IATs merely by being instructed to respond in a certain way. McDaniel et al. (2009) instructed participants to respond as extravertly as possible on an IAT which measured personality types and found that participants could successfully fake their level of extraversion. van Nunspeet et al. (2014) highlight a related finding where in an IAT to measure bias toward muslim women, framing the IAT task as a measure of competence (the participants ability to process new information) vs. morality (a test to measuring their “values”) resulted in reduced negative-bias IAT scores when the task was framed in terms of morality. Here they show how ERPs associated with early perceptual processing, selective attention and social categorization (namely N1, P150, and N450) are sensitive to this framing effect, further indicating the role of motivational states in modulating aspects of perceptual attention and conflict monitoring. In the next subsection we outline previous studies using EEG measures to study the IAT and in the following subsection we posit our research aims with respect to gaps in the existing studies.

### 1.1. Previous IAT-EEG work

Studies that have examined ERPs in IAT tasks (hereafter referred to as IAT-ERPs), have implicated the late positive potential (LPP) as a component of interest (e.g., Hurtado et al., 2009) or other later occurring (>300 ms) ERP components (e.g., O'Toole and Barnes-Holmes, 2009). Those focusing on both early and late activity, such as the study by Williams and Themanson (2011) investigating the IAT effect in a group bias IAT (gay-straight) reported no differences across IAT conditions for early components (N1, P2) but found later component differences (N400, LPP) for concept pairings. This is suggestive that early perceptual and attentional processes might not be associated with the IAT measurement in their study, but later semantic categorization processes are responding to congruent/incongruent concept pairings.

While many of the previous IAT-EEG studies examine ERP phenomena in response to the IAT task stimuli, others have explored ERP measures taken from separate (but related) tasks on the same participants in order to understand the time-course of neural processing of stimuli involved with implicit bias. He et al. (2009) demonstrated a relationship between the IAT effect on a racial IAT and early P2 and N2 components for categorization of faces (e.g., White, Black, Asian). Here they found correlations between ERP amplitudes in a racial face categorization task with an IAT-based measure of implicit racial bias in a group of non-muslim university students. Additionally, a later positive component (LPC) was observed for extended same-different race faces. Ibanez et al. (2010) has also shown that early ERP components of race-face processing (e.g., N170 component) can be modulated by the valence of evaluative attributes used in the IAT such as positive or negative valence words, and also by the social face categories such as in-group or out-group. This is suggestive of early integration of contextual information related to racial attitude during face processing in the IAT.

A recent study by Forbes et al. (2012) investigating EEG correlates of the IAT effect in an attempt to examine causal factors, showed more positive ERPs at frontal and occipital regions at automatic processing speeds, as well as occipital regions at controlled processing speeds, when responding to congruent vs. incongruent pairings. Here they investigated ERP timings as determinants of automaticy in order to gain insight into the timing at which implicit and explicit processes unfold, as these may be less susceptible (in short duration processes) to control. Moreover, they found higher D-scores (or bias) were identified by greater coherence between frontal and occipital regions in time periods as early as 92 ms with no significant difference present between congruent/incongruent conditions. These findings the authors consider could be indicative of top-down modulation of attention and perceptual processing. When taken in tandem with lesion study data they indicate the potential for the facilitated performance seen on stereotypic-congruent blocks to be associated with more efficient neural processing.

A number of other ERP components have been observed in the IAT-EEG such as the P3 which is associated with a range of cognitive processes, one of which is attentional focus on novel, salient or unexpected to-be attended items or on distractors (unattended items) which produce an orienting response (Polich, 2007). The P3 has also been shown to index explicit attention toward self-referent material (Tacikowski and Nowicka, 2010), and is also involved in implicit attention in an IAT toward self-positivity biased words (Chen et al., 2014).

Williams and Themanson (2011) demonstrate effects surrounding an N400 ERP where larger amplitudes are present in incompatible trials compared to congruent trials “suggesting greater semantic congruency in the compatible condition of the IAT.” They note N400 amplitude for both congruent and incongruent blocks at FCz is correlated with IAT incongruent-congruent reaction times with no apparent statistically strong relationships present with respect to reaction times in either block. The N400 ERP (as being distinct from the error-related N450; Folstein and Van Petten, 2008) is sensitive to semantic anomalies and violations with structures in the “immediate vicinity of the auditory cortex” (with a left-hemispheric dominance) associated with the processing of semantically anomalous sentences (Van Petten and Luka, 2006). While the N400 was initially thought to reflect linguistic anomalies and violations, further study has identified its role in semantic priming (Deacon et al., 2000) and expectancy (Curran et al., 1993). There is evidence that it too does not reflect a purely automatic process (Holcomb, 1988) involving attentional related factors. Lau et al. (2008) identify a dominant (left-hemispheric) pattern across a range of studies utilizing EEG and non-EEG imagining modalities investigating the N400, and indicate the posterior middle temporal cortex as being the only area to show consistent effects across studies.

A common finding among these studies is that both early and late time regions of the EEG signal following stimulus presentation, demonstrate effects related to IAT congruency condition and D-score. Earlier effects typically reflect neural mechanisms at work outside of a post-perceptual processing time region, namely one that occurs later within a time window following a response (Guex et al., 2011; van Nunspeet et al., 2014). There is no clear consensus on what ERPs and related morphologies should be found when examining a new IAT task. For instance, Fleischhauer et al. (2014) do not find evidence of significant effects in the N2 (as expected by the authors) or N400 time-range but do find relationships for P1/P3b amplitudes relating to early facilitation of relevant visual input and efficiency of stimulus categorization.

An open question remains from the literature as to what extent the IAT-effect can be modulated by external and other top-down related factors.

### 1.2. IAT and cognitive control

Hilgard et al. (2015) investigated the relationship between the medial frontal negativity (MFN) during an IAT task as it has been identified as linked to proactive cognitive control. This is distinct from a neurocognitive process of reactive control due to switching in incongruent and congruent block trials in the IAT task indicated by “a positive voltage deflection over frontocentral scalp locations” (D-pos). They posit their analysis and hypothesis in terms of a Dual Mechanisms of Control (DMC) model (Braver, 2012) where proactive control relates to “the sustained maintenance of goal information in working memory that serves to bias information in a goal-congruent manner” and reactive control “a late correction mechanism for dealing with cognitive and behavioral conflict as it arises.”

A common pattern of negatives have been commonly identified in IAT tasks in time regions related to the N2, N450, and related ERPs. Broadly, these ERP components are understood to be typically implicated in conflict monitoring processes including proactive and reactive cognitive control. Such negativities are often referred to as medial frontal negativities (MFNs). Identifying the onset/offset latencies of IAT-EEG sensitive ERP components like these is made difficult by their overlapping nature (variable latency), IAT-task parameters/stimulus affecting ERP waveform characteristics, the multifaceted nature of ACC-generated signals related to cognitive control and external (e.g., environmental) effects driving top-down attentional modulation.

An Error-related Negativity (ERN) typically follows post-error conflict detection between incompatible responses and the N2 has been found to reflect this monitoring and conflict detection function (Yeung et al., 2004). N2 amplitude is modulated by the amount of conflict present between possible choices prior to response selection and performance. Chee et al. (2000) used functional magnetic resonance imagery (fMRI) on participants completing an IAT and found that the left dorsolateral prefrontal cortex (dPFC) and the anterior cingulate cortex (ACC) mediate response inhibition in the incongruent condition. The ACC is particularly sensitive to response conflict in the IAT, and therefore N2 involvement in IAT performance at least ostensibly reflects conflict detection/cognitive control processes. Numerous other studies have implicated the ACC as being involved in the generation of a broad range of conflict-monitoring related ERP components (Bekker et al., 2005). Clayson and Larson (2013) demonstrated the N2 shows reliable conflict adaptation and these conflict adaptation indices were stable in a 2-week test-retest. Larson et al. (2014) highlight with regard to cognitive control theory and goal-directed behavior the N2 “represents an empirical marker of both a control mechanisms to handle conflict” and relevant to our study “a reflection of the level of cognitive control implemented during the …task.” They highlight other issues which can confound interpretations of N2 amplitude such as in flanker trials where N2 amplitude being “sensitive not only to the degree of conflict for a given stimulus but also to the extent to which task-irrelevant information is processed.” An important distinction between these studies (e.g., Eriksen flanker task) is that in IAT-EEG reactive control and conflict related changes arise as a result of task switching within condition blocks. Here, a participant is required to change between categorization of stimuli as evaluative (Nature, Built) or target categories (Me, other) with no knowledge of the upcoming trial type (Hilgard et al., 2015).

Given the overlapping time regions of these early negative components in existing IAT studies, one of the aims of our study introduced later was to disentangle ERP activity in these time windows using LORETA source analysis to explain patterns of correlated ERP activity with respect to implicated cortical generators of the N2/MFN and N400-related ERPs (cingulate cortex and temporal lobe structures).

This evidence would suggest ERP activity manifesting negatively in the 250–450 ms time range indexes a range of distinct neural processes related to cognitive control. Moreover, as activity in this time range is understood to be involved in proactive control processes, we posit the relationships observed to D-score (without an apparent explanation based purely on reaction time) may be indicating participant variability with regard to enhanced motivational/attentional aspects to perform the task “as quickly as possible,” thus engendering the measurement of an IAT-effect. Jodo and Kayama (1992) show that the N2 amplitude is enhanced by reaction time constraints in a go/no-go task varying in amplitude depending on the neuronal activity required for response inhibition, indicating increased amplitudes are related to a “greater effort” needing to be employed in tasks where response inhibition is constrained by reaction-time constraints. Such reaction-time constraints are integral to the measurement of the IAT effect where faster responding is presumed to be less susceptible to being faked.

Given evidence that groups typically have positive IAT scores on the n-IAT, we suspected those participants with lower D-scores (less standardized difference in reactions times) might be engaging in the task with different (less) motivational effort and consequently not engendering conditions needed to capture reaction time effects underlying implicit associations. Other authors highlight issues suggesting IAT effect measurement is potentially related to the “degree of involvement of the participants” (Vargo and Petroczi, 2013). Agosta et al. (2013) suggest a neutral D-score window for scores between −0.2 and 0.2 where results are “inconclusive” i.e., have low accuracy.

### 1.3. Aims and research questions

In summarizing previous related work on IAT-EEG issues we see that an open question exists as to what exactly the IAT measures given its susceptibility to be sometimes difficult to relate to explicit measures of attitude. In the study reported in this paper, we highlight a potential issue here, namely that successful measurement of an IAT effect likely involves factors of participant motivation to engage in the task such that some participants might be more likely than others to produce an IAT effect.

Accordingly, in this study we examine how ERP measures in the IAT might offer insight into the neural mechanisms underlying the more rapid associations that drive IAT effects. Of primary interest to our work was examining how ERP measures underlying both congruent and incongruent block types could offer evidence on the neural mechanisms underlying more rapid associations driving the IAT effect. Our hypothesis is that such a shared relationship would exist, further implicating proactive cognitive control and top-down modulation of attention/sensory processing (involved with potential motivational factors) in biasing the IAT measurement.

In summary, the aim of our study was (1) to examine how ERP measures in the IAT might offer insight on the neural mechanisms underlying the more rapid associations that drive the IAT effect and (2) given such relationships, to investigate potential sources of correlated brain-activity using LORETA as previous IAT research has implicated a range of early negativities overlapping in time and scalp topography serving arguably different processes in the IAT.

## 2. Materials and methods

### 2.1. Participants

Thirty participants aged between 18 and 45 years were recruited through advertisement using Dublin City University staff and undergraduate/postgraduate email lists. In total, 8 participants were excluded due to noisy EEG, high error response rates, or to ensure a counter-balanced block design for the order of congruent/incongruent IAT blocks (i.e., same number of congruent first and incongruent first block orderings). The 22 remaining participants were predominantly right handed.

### 2.2. Approval by university ethics committee

This study was carried out in accordance with the Declaration of Helsinki—Ethical Principles for Medical Research Involving Human Subjects, and also Dublin City University's guidelines on Best Practice in Research Ethics with informed written consent from participants. The study was approved by Dublin City University's Research Ethics Committee (DCU REC/2013/205). All participants gave written informed consent.

### 2.3. Implicit association task

Participants in this study completed a modified version of the IAT outlined by Bruni and Schultz (2010). The purpose of this was to measure the strength of the association between the “Me” target category and two evaluative attribute categories (“Nature,” “Built”) relative to the associative strength of the “Other” target and attribute categories. The experiment consisted of 7 blocks with 2 blocks (of 32/48 trials, respectively) measuring congruent association RTs (e.g., “Nature-Me,” “Built-Other”), and another 2 blocks (of 32/48 trials, respectively) measuring incongruent association RTs (e.g., “Nature-Other,” “Built-Me”). The remaining blocks were practice blocks. Congruent/Incongruent ordering was counter-balanced across participants. In total, 80 trials were collected for each of the congruent and incongruent mappings, respectively.

Participants were required to sort stimuli into category pairings of “Me-Nature” “Other-Built” in the congruent case and “Me-Built” “Other-Nature” in the incongruent case. Each participants' name was used in conjunction with the “Me” category and a random list of other names for the “Other” category. “Tree,” “Mountain,” “Butterfly,” and “Flower” were used as stimuli for the “Nature” category. “Boat,” “Car,” “Chair,” “Truck” were used as stimuli for the “Built” category. Category pairings (e.g., “Me-Nature, Other-Built”) appeared in the upper left and right corners of the screen, respectively. Participants indicated the category to which stimuli belong by pressing keys “1” (pairing appearing on left side of screen) or “2” (pairing appearing on right side of screen) on the keyboard with their dominant hand.

**Figure 1:**
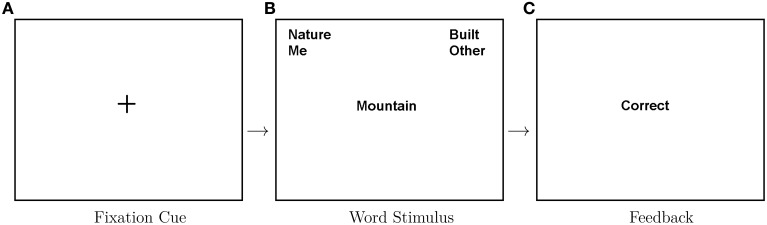
**Depiction of trial structure within a congruent block**. From left to right: a fixation cross is presented on screen for 1 s, then a word is presented for categorization and following a key press a feedback screen is presented for 1 s indicating whether the response was correct. Eighty were recorded for each congruency condition.

Stimuli were preceded with a 1 s central fixation located centrally on-screen. A word stimulus was presented on screen (centrally) until a response was given. A feedback screen appeared post-response based on correct/incorrect categorization. Participants were not required to correct response errors. In Figure 1 we show an example of this trial structure within a compatible (congruent)-mapping block.

D-score was calculated for each participant as the difference in mean reaction time between trials from pooled incongruent and pooled congruent blocks (incongruent-congruent) divided by the pooled standard deviation of trials from both block types (Greenwald et al., 2003).

### 2.4. EEG recording and analysis preliminaries

EEG was recorded using a 32-channel ActiCHamp recording system with a 10–20 ActiCap. A virtual ground was used as an online reference and later re-referenced offline to a digitally linked-mastoids reference (TP9 + TP10). Prior to this, signals were filtered with an FIR sinc hamming window filter to between 0.1 and 30 Hz. ICA (Independent Component Analysis) was used to remove artifacts such as eye-blinks and eye-blink related components in particular those described by Plöchl et al. (2012) as CRD (corneo-retinal dipoles), eyelid and eyelid-CRD artifacts. These were identified from scalp topography and amplitude characteristics and similarily confirmed using EOG (Electrooculogram) channels VEOG and HEOG. ICA weights were trained on 1–30 Hz filtered data and then applied to the 0.1–30 Hz band-passed signals. Analysis revealed strong pre-stimulus activity related to block conditions. Subsequently, ERP averages were generated on epochs extracted from signals band-passed to between 4 Hz and 30 Hz (we explain this in the next section). Epochs were extracted from −200 to +1000 ms with respect to the onset of word stimuli to be categorized for compatible and incompatible blocks. Trials (post ICA clean-up) which exceeded 70 mV or contained other noise-like artifacts were discarded. This resulted in a maximum of 7.5% trial loss across participants with one subject exceeding this at near 20%.

EEG recording was carried out in an electrically shielded environment. Participants were seated approximately 70 cm from the screen and reported no issues reading word associations.

EEGLAB 13.32 was used for EEG pre-processing and clean-up. Scalp and statistical scalp plots including grand average ERP plots were generated in IPython. SPSS 21 was used for conducting repeated-measures ANOVA. sLORETA 20081104 was used for source localization measures (Pascual-Marqui, 2002).

#### 2.4.1. Baselining

A baseline of −200 s to 0 pre-stimulus was initially planned for stimulus-locked epoch extraction. However, upon analysis of the EEG it was found that a CNV (Continent Negative Variation)-like component was present surrounding stimulus onset for many participants, and upon further inspection differentiated between congruent and incongruent conditions.

Baselining serves to remove noise sources like inter-subject differences and slow-drifts, thus allowing inter-subject measures to be comparable as ERP amplitudes align relative to a zero measure (baseline) across electrodes and participants. An expectation here is that pre-stimulus baseline activity is not systematically affected with respect to factors or conditions being measured. Herein the issue exists with the IAT experimental structure, that is, a participant is aware of the upcoming condition type and thus may, through the recruitment of different cognitive preparatory mechanisms for that stimulus type (congruent/incongruent), introduce into the baseline period activity which could systemically affect the correct baselining of later ERP components. This is particularly relevant as pre-stimulus activity diminishes during the epoch window. One such ERP component typically seen, a CNV-E (Contingent Negative Variation), is present following a warning stimulus (S1) such as a fixation cross indicating upcoming stimulus (S2) and results in an expectant pattern of activity locked to S2.

In our study, when examining ERP activity in these early time regions in individual participants' data plots (without baselining across a range of incrementally high-pass filtered signals) we found there was a general trend of pre-stimulus activity extending into early periods of the ERP waveform, overlapping notably with the P1. Other IAT-ERP studies might not have considered or encountered such issues with some studies not citing whether a baseline was used (Barnes-Holmes et al., 2004; Hurtado et al., 2009; Egenolf et al., 2013), others where a prestimulus baseline was used (O'Toole and Barnes-Holmes, 2009; Williams and Themanson, 2011; Hilgard et al., 2015) and others where a prestimulus baseline was used but measures were taken to lessen the impact of pre-stimulus activity such as post-movement ERP activity related to previous trials Forbes et al. (2012).

We found ERP average waveforms from participants without pre-stimulus baselining indicate these differences in some instances do not degrade until 150 ms and would suggest these differences, if included in the baseline measurement, could systematically affect later ERP components, resulting in these component time-windows containing ostensible effects.

Time-frequency decomposition of epochs and related ITC (Inter-Trial Coherence; Makeig et al., 2002), revealed that pre-stimulus activity is comprised of contributions across a wide range of frequencies. Inspection of stimulus-locked ICA components revealed these patterns are not well captured by a single set of ICs that are not entangled with other post-stimulus trial-locked ERP-related activity. Ultimately we felt this precluded us from meaningfully interpreting earlier ERP component time-windows that overlap with this potential systematic bias. Later ERP components are subsequently increasingly affected if a pre-stimulus baseline is used if differentiating CNV activity stemming from condition type (congruent vs. incongruent) is present during this baselining period.

One strategy to reduce confounding systematic differences in this late CNV component is not to allow the participant to be aware of the upcoming stimulus type/task, that is, by not having blocks with consistent conditions allowing for different neural preparatory mechanisms to affect pre-stimulus time regions where baselines are typically extracted from. This strategy would deviate somewhat from the typical IAT task structure as it would require adding another dimension of task switching in the IAT (compared to just between attribute and target categories). Furthermore, in this instance at the time of stimulus onset, a participant would need to be aware of the condition type, thus further introducing deviations of the IAT experiment structure. Merely changing the corner labels to inform the participant would not likely be perceptible until foveation, further introducing confounds related to required eye-movements and very likely degrading time-locking characteristics of the ERP components being studied. Other strategies include varying the S1–S2 difference timings to mitigate consistent pre-stimulus-locked activity but this process may merely serve to obscure the level to which preparatory-related EEG signals and other time-locked within-block ERP activity might be affecting baselines.

A primary reason for baselining is to remove slow drifts present in the EEG, which when removed by high-pass filtering can result in obscured ERP amplitude/latency characteristics (Rousselet, 2012), particularly so when the ERP is generated as a result of lower frequency band activity. An issue with this is comparability with other studies as some ERP components may have lost contributing stimulus-locked and phase-coherent related activity in lower frequencies. However, doing this allows us to overcome some of the problems for which we use baselining in the first place, that is to remove slow drifts and other noise sources which complicate comparison of ERP amplitude activity across participants.

The restrictions imposed by the IAT experimental design give rise to a number of confounds when adopting a typical ERP processing strategy. This does not reflect a fundamental flaw in the IAT task itself, but rather a characteristic of it that does not fit the typical ERP processing pipeline. In this study we high-pass filter EEG signals in order to overcome these limitations at some cost to the comparability of amplitude/time characteristics to other EEG studies. By not doing so, however, we introduce systematic confounds across the analysis time-window. Examination of the impact of this high-pass filtering on ERP waveforms would indicate it is largely non-detrimental to activity in early ERP-component time-windows (N1, P2, N2, P3) but does largely affect (attenuate) stereotyped late P3b activity, which is notably comprised of lower EEG frequencies in the delta band (0–4 Hz; see Demiralp et al., 2001b). We focus our analysis on time regions where activity related to N1, P2, N2, and early P3 contributions are present.

Bidet-Caulet et al. (2012) outline similar issues encountered pre-stimulus with regard to the baselining and CNV activity, and they use an approach of high-pass filtering at 4 Hz in order to effectively analyze early ERP stimulus-locked ERP components.

ERP plots (including a range of other scalp plots and graphs) used in this study are given in the supplementary data for this paper using a variety of frequency and baselining methods to further highlight this problem and how our solution results in earlier ERP waveform characteristics being largely retained, both in amplitude and timing. Time-frequency wavelet analysis too indicates there are different frequency and spatial topographies for the ERP components of interest and their correlative relationship to D-score, indicating high-pass filtering artifacts do not contribute to this result.

#### 2.4.2. Electrode reference choice

Other ERP studies investigating the IAT have typically used an averaged (linked) mastoid reference (TP9 + TP10). EEG reference choice is known to affect the spatial, temporal and polarity characteristics of ERP waveforms and hence the chosen reference site should be carefully considered not only to allow comparability of results to other studies but also such that it is not affected by activity related to the factors being investigated in the experiment.

The spatial dispersion of statistical activity seen in statistical scalp plots in our study suggests that the linked-mastoid reference choice may not be entirely optimal and should at least warrant consideration as these electrode sites are located near to temporal-lobe regions implicated in language-processing. While there is generally high agreement in our study for the locus points of statistical activity between linked-mastoid and common average reference schemes, differences are evident notably in terms of higher spatial dispersion of statistical activity for the linked-mastoid reference to a common average reference scheme. In the supplementary appendix to this paper, we provide grand average ERP waveforms using a common average reference scheme to highlight the potential contribution of activity at the TP9 and TP10 reference sites. Similar issues surrounding EEG referencing schemes are also considered by Dien (1998), Hagemann et al. (2001) and Luck (2005).

### 2.5. Analysis of neural data

#### 2.5.1. ERP time-windows and channel selection

ERP time-windows were determined by inspecting grand-averaged ERP plots across participants irrespective of condition type. ERP time-windows were selected so as to include within the window a primary, and any secondary, troughs or peaks characteristic of ERP activity of that type. An important point to note is that peaks in ERP averages are not the same as ERP components, as ERP components contributing to averaged activity can have varying latencies and overlap. In this work we refer to ERP time-windows as time periods known to contain stereotyped underlying ERP activity. A further discussion of this can be found in Luck (2005).

There are topographic variations of ERP activity in the IAT literature implicating a number of fundamental ERP components active in time regions corresponding to the P1, N1, P2, N2, and P3. To our knowledge, as we are first to investigate ERPs in the nature-IAT. We did not preselect explicit channel-ERP mappings in our study. Instead, we identified these channels and time regions from visual inspection of ERP time-topographies on grand-averaged epochs—averaging across participants and conditions. With respect to time regions and channels, the literature identifies a variety of stereotyped ERP morphologies that can be present in the IAT. Importantly here, there are variations in expected ERP channel × time morphologies determined by the IAT task itself and the stimulus content used (pictures vs. words) introducing uncertainty with regards to what the expected ERP patterns will be in an untested IAT.

In our study, the N100 was identified as being present in the 110–150 ms time window, the P200 in the 160–230 ms time window, a pattern of fronto-central tending negativity hereafter referred to as N200 in the 250–310 ms time window and a frontal P300-like component in the 330–450 ms time-window.

From the existing IAT (and EEG) literature we know a broad range of ERP components are to be expected such as the P1, N2, P2, P3, and N400 in the IAT-EEG. From N200 studies, we know variations of this component can manifest with anterior (Fz), central (Cz), and posterior (Pz) scalp distributions. Similarly, N400 ERP effects are described occurring in overlapping time periods on these electrode locations. Given our focus investigating early negative ERPs (N2, N400, MFN) electrode sites Fz, Cz, and Pz were chosen as *regions of interest* (ROIs) with regard to the IAT and key electrode sites for comparisons.

#### 2.5.2. Repeated-measures ANOVA

Repeated-measures ANOVAs were used to identify significant neural activity during ERP time regions. Channels for each repeated-measures ANOVA were identified from grand-average ERP plots without differentiating trials based on D-score type or condition (congruent/incongruent), selecting those that displayed stereotyped ERP activity of the N1, P2, N2, and P3.

Repeated-measures ANOVA models were used for each identified ERP time frame examining electrode site × condition (Congruent/Incongruent) as within-participant factors and a between-participant factor of “D-score range” identifying high, medium and low D-scorers (a 7/8/7 split, 22 in total). Greenhouse-Geisser corrected *p*-values and statistics are reported.

#### 2.5.3. Repeated-measures ANOVA *post-hoc* analysis

Correlation based measures are used as part of our *post-hoc* RM-ANOVA analysis given the presence of between-subject effects of D-score magnitude. These are presented both in terms of contrast, explaining significant effects found in our ANOVAs and in parallel as measures to capture a type of statistical relationship not readily captured by repeated-measures ANOVA analysis.

Correlations are examined using EEG time-window average amplitudes. In **Table 2**, we show Pearson-r correlational coefficients for behavioral measures and ERP time-window activity across selected electrode sites Fz, Cz, and Pz, and for electrodes of peak correlation.

#### 2.5.4. The LORETA approach

sLORETA is used alongside correlation analysis with D-score, to identify potential functionally and spatially distinct brain regions that are active in ERP time ranges. Given the complexity of the resulting relationships, either temporal or spatial in nature, which are introduced by utilizing reference channels that are not electrically silent (i.e., located near to language areas), scalp plots of ERP or statistical activity can be misleading as activity at a particular site might be indicative of two or more channels (and/or ERP components) interacting in a complex way.

In this study, LORETA is used to identify, within the precision of LORETA's localization error, brain regions and structures involved with early ERP component activity which gives a better sense of cortical regions that are involved. Both approaches are carried out here as they are considered complimentary in understanding brain activity driving early IAT-ERP effects.

Reported LORETA correlation *p*-values are adjusted for multiple comparison and presented in the format [*r* = 0.51, *p* = 0.005].

### 2.6. Conventions used in the analysis description

Further references to congruent and incongruent EEG and reaction times will be described in a format of *measure-type(measure-src)*: *RT*(*C*) = congruent reaction time, *RT*(*I*) = incongruent reaction time, *RT*(*I* − *C*) = *RT*(*I*) − *RT*(*C*), *E*(*C*) = congruent EEG amplitude measure, *E*(*I*) = incongruent amplitude measure and *E*(*I* − *C*) = *E*(*I*) − *E*(*C*).

Significant trends are reported for *alpha* < 0.05 and weakly significant trends for *alpha* < 0.10.

Statistics for both multivariate and univariate are reported inside square brackets e.g., [*r*_(21)_ = 0.8, *p* = 0.001].

#### 2.6.1. Other methods

There is evidence for the presence of non-linear relationships surrounding ERP measures with regard to IAT-effect in our experiment as has been found in other studies Williams and Themanson (2011). Although we do not explore these relationships in the paper, we include them in the Supplementary Materials.

## 3. Results

### 3.1. Behavioral IAT analysis

Analysing the behavioral RT data for participants between congruent (*M* = 731.73 ms, s.e. = 296.13) and incongruent (*M* = 822.96 ms, s.e. = 338.2) conditions, there was a significant difference found in reaction times. Reaction times for each condition for each subject submitted to a Wilcoxon signed-rank test revealed significant differences in reaction time [*Z* = 19, *p* = 0.000483]. This confirms our group shows a pro-nature bias.

**Figure 2:**
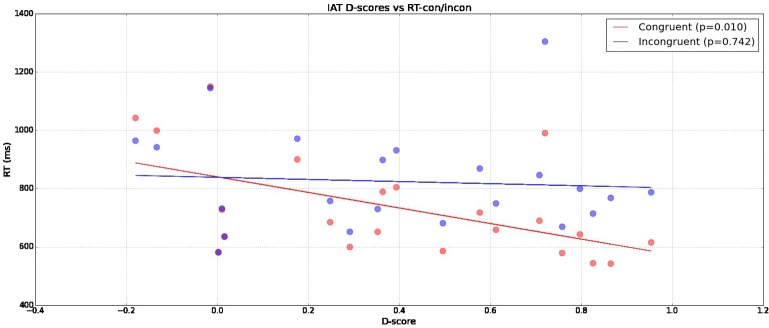
**Reaction times across subjects broken down across congruent and incongruent conditions (y-axis) with calculated D-scores (x-axis)**.

In Figure 2 we can see that a significant correlation exists between a participant's D-score and reaction time in congruent (Pearson-r *p* = 0.01023) conditions compared to incongruent (Pearson-r *p* = 0.74158) conditions. This indicates our measured IAT-effect is being driven by reduced reaction times in congruent blocks without corresponding related increases in incongruent block reaction times.

### 3.2. Neural IAT analysis (ANOVA)

#### 3.2.1. Repeated measures ANOVA analysis

Amplitude averages across participants for ERP time-windows were submitted to a repeated measures ANOVA with congruency conditions and channels as within-subject factors, and D-score range as a between-subject factor. D-score ranges were acquired by using a 7/8/7 split (by D-score) of available participants. Effects with a significance of *alpha* < 0.10 are reported. In Figure 5 we show ERP averages across condition, D-score range and electrode site.

##### 3.2.1.1. N100

The N100 was examined across electrode sites Fz, Cz, Pz, F3, F4, C3, C4, P3, P4, CP1, CP2, FC1, and FC2. A significant main effect was found for channels $\left[F_{\left(2.914,55.357\right)}=37.682,\eta^{2}=0.665,p<0.001\right]$.

##### 3.2.1.2. P200

The P200 was examined across electrode sites Fz, Cz, F3, F4, C3, C4, FC1, and FC2. A significant main effect for channels was found $\left[F_{\left(2.562,48.683\right)}=35.202,\eta^{2}=0.478,p<0.001\right]$.

##### 3.2.1.3. N200

The N200 was examined across electrode sites Fz, Cz, Pz, F3, F4, C3, C4, P3, P4, CP1, CP2, FC1, and FC2. Main effects were found for channels $\left[F_{\left(2.588,49.171\right)}=24.279,\eta^{2}=0.561,p<0.001\right]$, conditions $\left[F_{\left(1,19\right)}=3.252,\eta^{2}=0.146,p=0.087\right]$ and D-score range $\left[F_{\left(2,19\right)}=4.866,\eta^{2}=0.339,p=0.02\right]$. A weakly significant interaction effect for condition × D-score range was found $\left[F_{\left(2\right)}=1.34,\eta^{2}=0.124,p=0.079\right]$.

##### 3.2.1.4. P300

The P300 was examined across electrode sites Fz, F3, F4, FC1, FC2, Pz, P3, P4, C3, C4, Cz, CP1, CP2, CP5, and CP6. Main effects were found for channels $\left[F_{\left(2.580,49.023\right)}=15.586,\eta^{2}=0.451,p<0.001\right]$ and D-score range $\left[F_{\left(2,19\right)}=7.529,\eta^{2}=0.442,p=0.004\right]$. No main effect was found for congruency condition.

### 3.3. Neural correlates of D-score

#### 3.3.1. N100, P200

As neither the N100 or P200 time windows emerged with significant effects (i.e., *p* < 0.10) we do not report them further in this study.

#### 3.3.2. N200

Repeated-measures ANOVA revealed a number of significant effects for the N200 for between-subjects (i.e. D-score is predictive of ERP amplitudes) and of within subject-effects, such that N2 amplitudes congruent (*M* = −1.983 mV, s.e. = 0.274) were enhanced (more negative) compared to incongruent (*M* = −1.798 mV, s.e. = 0.25) conditions. There was an effect for between-subjects for D-score range indicating mean amplitudes were more negative for high D-scores (*M* = −3.037 mV, s.e. = 0.456) compared to low D-scores (*M* = −1.339 mV, s.e. = 0.456) and different form medium D-scores (*M* = −1.295 mV, s.e. = 0.426).

Condition × D-score emerged as a significant interaction where medium D-scores displayed greater mean amplitude differences between congruent (*M* = −1.537 mV, s.e. = 0.486) and incongruent conditions (*M* = −1.053 mV, s.e. = 0.414) compared to differences between conditions for high D-score congruent (*M* = −2.978 mV, s.e. = 0.486) and incongruent (*M* = −3.095 mV, s.e. = 0.443), and low D-score congruent (*M* = −1.433 mV, s.e. = 0.486) and incongruent (*M* = −1.245 mV, s.e. = 0.443) conditions.

Significant linear relationships were present for the N200 time-window examining Pearson-r correlation between D-score congruent [CP6, *r* = −0.54, *p* = 0.009] and incongruent conditions [C4, *r* = −0.54, *p* = 0.009] (**Table 2**).

Examining **Table 2** we see see these linear relationships are primarily constrained with respect to D-score × amplitude with no significant (univariate) correlations present at electrode sites (matched for the electrode site with the most significant correlation) comparing other behavioral measures. Here we can see stronger patterns of correlation across electrode sites for congruent reactions times to neural measures than incongruent reactions times. Similarly, we see increased correlations for the standardized reaction differences between congruent blocks (D-score) compared to non-standardized differences [i.e., rt(I-C)].

**Figure 3:**
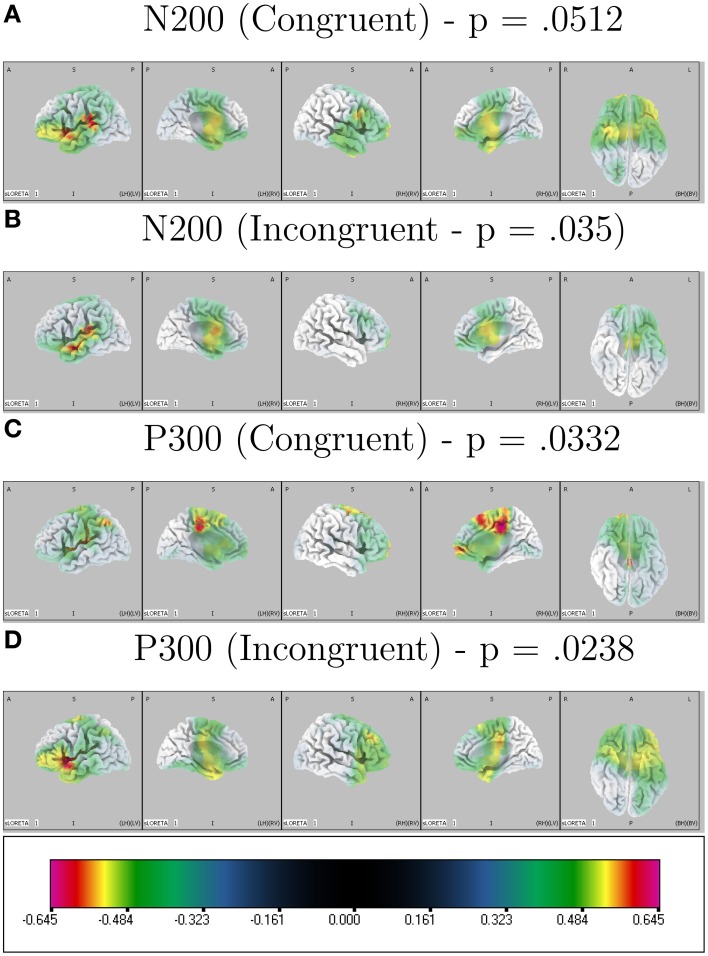
**Correlated LORETA voxel activity and D-score**. D-score is correlated with congruent and incongruent ERP time-window averages across participants localizing activity driving correlated scalp EEG measures. Multiple comparison corrected *p*-values for peak correlations are presented on top of each condition × ERP plot.

**Figure 4:**
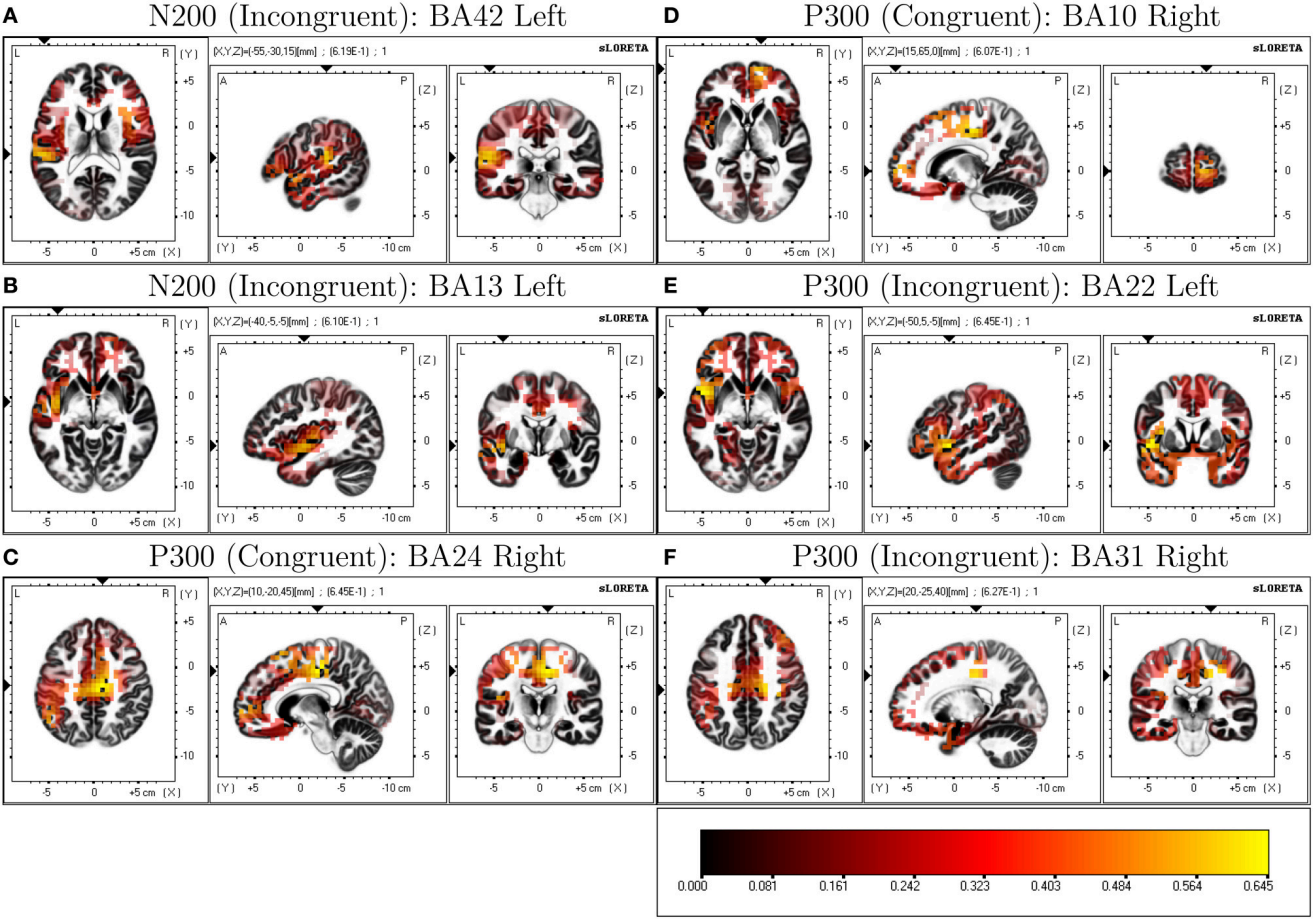
**Selected ROIs revealed through LORETA D-score regressions**.

LORETA analysis shown in Figures 3A,B reveals characteristic shared activations between congruent and incongruent conditions in similar brain structures with these outlined in Table 1 and Figure 4. Broadly, most significant correlations with D-score were found in areas extending from anterior, inferior, and insular regions of the left temporal lobe (BA13) and postcentral gyrus (BA 43) as shown in Table 1 (BA42, BA13, BA43, and BA22).

**Table 1:** **LORETA-derived regions of peak correlation of D-score across congruent and incongruent conditions**.

**Component**	**Condition**	**Area**	**Brodmann area**	**Side**	***R***	***P***	***X***	***Y***	***Z***
N200	C	Temporal lobe - STG	42	L	0.619	0.0530	−55	−30	15
		Temporal lobe - STG	22	L	0.6	0.0734	−45	5	−5
		Insular - Sub-lobar	13	L	0.595	0.0786	−40	5	−5
		Postcentral gyrus^*^	43	L	0.598	0.075	−65	−20	20
		Insular - Sub-lobar^*^	13	L	0.550	0.144	−45	0	−10
	I	Postcentral gyrus	43	L	0.643	0.041	−65	−20	20
		Insular - Sub-lobar	13	L	0.605	0.0812	−45	0	−10
		Temporal lobe - STG^*^	42	L	0.541	0.190	−55	−30	15
		Temporal lobe - STG^*^	22	L	0.521	0.230	−45	5	−5
		Insular - Sub-lobar^*^	13	L	0.560	0.149	−40	5	−5
P300	C	Cingulate gyrus	24	R	0.645	0.033	10	−20	45
		Insular - Sub-lobar	13	L	0.623	0.0548	−45	−25	20
		Medial frontal gyrus	10	R	0.605	0.0732	15	60	5
		Superior temporal gyrus^*^	22	L	0.534	0.1842	−50	5	−5
		Cingulate gryus^*^	31	R	582	0.103	20	−25	40
		Postcentral gryus^*^	3	R	0	0.99	30	−25	40
	I	Superior temporal gyrus	22	L	0.645	0.0244	−50	5	−5
		Cingulate gryus	31	R	0.627	0.037	20	−25	40
		Postcentral gryus	3	R	0.627	0.037	30	−25	40
		Cingulate gyrus^*^	24	R	0.570	0.095	10	−20	45
		Insular - Sub-lobar^*^	13	L	0.501	0.231	−45	−25	20
		Medial frontal gyrus^*^	10	R	0.459	0.333	15	60	5

Rows marked with

* *are provided to allow comparison of matched MNI (x, y, z) coordinates between respective maximima of peak correlation between congruent and incongruent conditions*.

**Figure 5:**
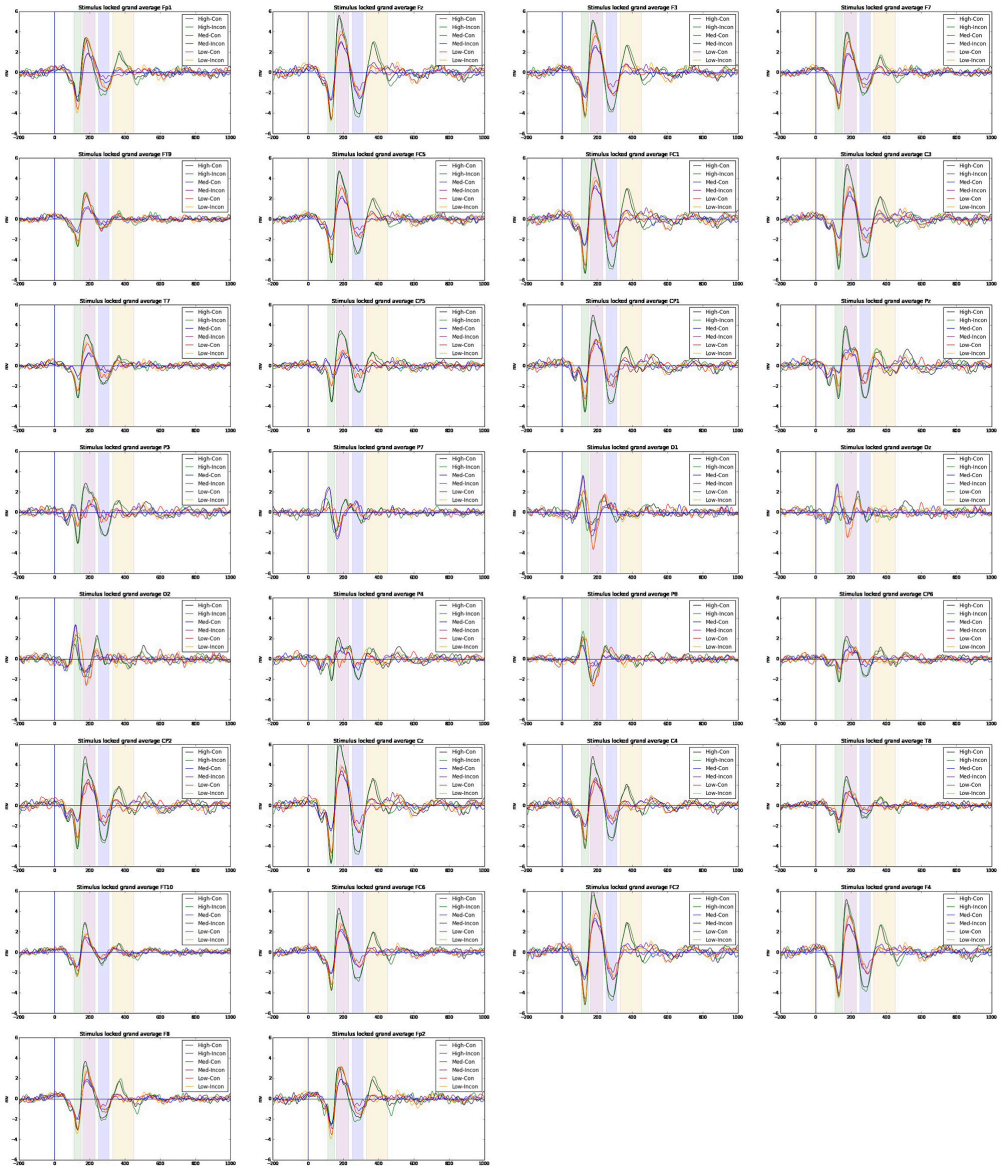
**ERP averages across electrode sites for high, medium and low D-scorers across congruent/incongruent conditions using a linked-mastoids reference**. Signals are filtered in the range 4–30 Hz.

#### 3.3.3. P300

Repeated-measures ANOVA for the P300 time-window revealed a significant relationship for a between-subjects effect for D-score range such that mean ERP amplitudes were larger in high (*M* = 0.763 mV, s.e. = 0.140) compared to medium (*M* = 0.119 mV, s.e. = 0.131) and low (*M* = 0.087 mV, s.e. = 0.130) groups.

LORETA revealed significant patterns of activation revealed as correlated with D-score for congruent and incongruent conditions. Figures 3C,D show the respective congruent and incongruent correlated activations.

More prominent differences emerge, differentiating correlated neural activity of the congruent and incongruent conditions for regions surrounding: Medial Frontal Gyrus (BA 10) being more correlated with D-score in congruent [*r* = 0.605, *p* = 0.0732] v. incongruent conditions [*r* = 0.459, *p* = 0.333], and for Postcentral Gyrus (BA 3) in congruent [*r* = 0.0, *p* = 0.99] v. incongruent conditions [*r* = 0.627, *p* = 0.037] (Table 2).

**Table 2:** **Pearson-r correlation coefficients across behavioral and EEG activity measures**.

	**N200**				**P300**			
	**Fz**	**Cz**	**Pz**	**Max**	**Fz**	**Cz**	**Pz**	**Max**
D,I-C	−0.21	−0.14	−0.08	0.39^**^(*P*8)	0.35	0.40^**^	0.50^*^	0.50^*^(*Pz*)
D,C+I	−0.46^*^	−0.47^*^	−0.42^**^	−0.54^*^(*C*4)	0.57^*^	0.52^*^	0.34	0.62^*^(*C*4)
D,I	−0.48^*^	−0.49^*^	−0.44^*^	−0.54^*^(*C*4)	0.61^*^	0.57^*^	0.47^*^	0.64^*^(*C*4)
D,C	−0.44^*^	−0.45^*^	−0.39^**^	−0.54^*^(*CP*6)	0.51^*^	0.42^**^	0.13	0.54^*^(*F*4)
rt(I-C),I-C	−0.17	−0.11	−0.04	0.36^**^(*P*8)	0.29	0.34	0.37^**^	0.37^**^(*Pz*)
rt(I-C),C+I	−0.26	−0.32	−0.36	−0.38^**^(*C*4)	0.51^*^	0.46^*^	0.37^**^	0.57^*^(*C*4)
rt(I-C),I	−0.29	−0.33	−0.37^**^	−0.39^**^(*C*4)	0.55^*^	0.51^*^	0.44^*^	0.60^*^(*C*4)
rt(I-C),C	−0.25	−0.30	−0.34	−0.41^**^(*CP*6)	0.48^*^	0.39^**^	0.22	0.50^*^(*F*4)
rt(C),I-C	0.14	0.15	0.10	−0.09(*P*8)	−0.11	−0.10	−0.17	−0.17(*P*z)
rt(C),C+I	0.35	0.36^**^	0.35	0.27(*CP*6)	−0.32	−0.33	−0.13	−0.28(*F*4)
rt(C),I	0.35	0.37^**^	0.36^**^	0.40^**^(*C*4)	−0.30	−0.30	−0.15	−0.27(*C*4)
rt(C),C	0.33	0.32	0.30	0.23(*CP*6)	−0.29	−0.30	−0.03	−0.24(*F*4)
rt(I),I-C	0.05	0.10	0.09	0.11(*P*8)	0.04	0.08	0.03	0.03(*Pz*)
rt(I),C+I	0.22	0.20	0.16	0.19(*C*4)	−0.05	−0.09	0.07	0.01(*C*4)
rt(I),I	0.21	0.20	0.17	0.19(*C*4)	−0.01	−0.03	0.08	0.05(*C*4)
rt(I),C	0.21	0.17	0.13	0.01(*CP*6)	−0.04	−0.09	0.09	0.02(*F*4)

*Correlated variable pairs presented in the first column as Behavioral Measure, EEG Measure*.

* Indicates univariate p < 0.05 and

** Indicates univariate p < 0.1. D, D-score;

*C, Congruent; I, Incongruent; rt(), Reaction Time. Max columns represent the electrode site with smallest p-value for D-score correlated with EEG measure (first 4 rows) where electrode site for each EEG measure type (C+I, I-C, C, I) is maintained across subsequent comparisons as a way to interpret the source of EEG activity driving correlations with D-score at that site*.

Non-standardized reaction time differences here [rt(I-C)] seem to show increased patterns of correlation to neural measures respective to D-score (compared to the case for the N2) suggesting that differences in reaction times (related to response-locked activity) are more likely driving contributions here for the IAT measurement.

## 4. Discussion

The aim of the study reported here was to examine how ERP measures in the IAT might offer an insight into the neural mechanisms underlying the more rapid associations that drive IAT effects. Of primary interest in our work was examining how ERP measures underlying both congruent and incongruent block types might offer evidence of the neural mechanisms involved with these relatively more rapid associations. In our results from behavioral measures we find average congruent reaction times are significantly correlated with participant D-score, while the reaction times in incongruent conditions are not. From this we would expected neural activity predictive of D-score to be present only in congruent blocks. Similarly, we would expect ERP measures for time-windows during the incongruent blocks to be largely unpredictive of D-score, however, we find this is not the case.

Our hypothesis was positioned such that in a situation where the measurable IAT effect is primarily modulated by reduced congruent reaction times, in the respective incongruent blocks we should find shared patterns of ERP activity correlated with the size of IAT effect, given the involvement of proactive cognitive control and other top-down control processes. This is related to the motivational/attention aspects in the IAT affecting the level to which an implicit bias might be measured. Given evidence that groups typically have positive IAT scores on the n-IAT, we suspected those participants with lower D-scores (a lower standardized difference in reactions times) might be engaging in the task differently due to factors like less motivational effort thus not engendering conditions necessary to capture IAT effects.

The aim of searching for such evidence was to disentangle cortical generators involved with the production of an IAT effect in early time periods of the ERP time window (early negativities between 250 and 450 ms) following stimulus presentation that have been previously implicated in other studies to be sensitive to the IAT effect size, and in cognitive control and error monitoring.

In our study we identified an N2-like ERP component in the 250–310 ms range. While we have labeled activity in this time-region as indicative of an N2 ERP, there is close overlap in time regions of an N400 described in other related EEG-IAT studies. Importantly, some of these studies identify correlational relationships between congruent, incongruent and incongruent-congruent activity ERP measures and D-score (Williams and Themanson, 2011). The N400 has been widely used as a measure of semantic congruency for words (Kiefer, 2002) and statements (Kutas and Hillyard, 1980). Williams and Themanson (2011) report a significantly smaller N400 for congruent conditions compared to incongruent conditions in an IAT suggestive that the N400 is an indicator of semantic (integration) congruency where greater incongruency results in larger (more negative) amplitudes. LORETA analysis estimating the source of correlated neural activity and D-score for both block types in our study implicate a number of left-temporal cortical regions, known generators in the N400 and more widely understood to be involved with language processing (Maess et al., 2006). Lau et al. (2008) identify a dominant (left-hemispheric) pattern across a range of studies utilizing EEG and non-EEG imaging modalities investigating the N400, and indicate the posterior middle temporal cortex as being the only area to show consistent effects across studies. It would seem that although no apparent N400 ERP component is present in our averaged waveforms, there is evidence overlapping ERP activity from the N400 time-frame might present during our N200 analysis window.

Forbes et al. (2012) suggest that a number of brain regions surrounding the left temporal lobe (as indicated by integrating both EEG source localization and lesion studies) are implicated as being important in the production of reduced congruent reaction times in the IAT. Interestingly, they find patients (lesion) vs. controls show no significant difference on incongruent reaction times or D-scores but show statistically significant differences where patients were slower to respond in congruent conditions. Similarly, they identify that volume loss in large regions of the left insula exhibit robust associations with slower reaction times in congruent blocks. In the context of our results, these findings support the role of left temporal/insular brain regions as being important in the production of an IAT effect. Another similarity in results is a strong indication that a number of shared brain structures are recruited across both congruent and incongruent conditions, but importantly there are differences associated with activations, suggesting different recruitment of brain regions based on condition.

The N2 has been found to reflect a conflict detection function (Yeung et al., 2004) between possible choices prior to response selection and performance. Hilgard et al. (2015) show that the medial-frontal negativity (MFN) ERP between 250 and 450 ms post stimulus at midline regions is larger for incongruent mappings, compared to congruent mappings, indicating increased proactive control is required during incongruent blocks. Although we find congruent (instead of incongruent) mappings in our IAT generated seemingly more negative going waveforms in this time region, their study highlights how the involvement of reactive control in the IAT (due to task switching) generates a similar temporal and spatially overlapping positive ERP (D-pos) in this time region which might be one explanation for the relationship we found. Importantly, as relative differences in these ERP measures between congruency conditions have been to understand congruency effects with respect to semantic integration and cognitive control, we find absolute measures here to be involved as well. There is other evidence that for early ERP negativities being sensitive to task constraints, for example Jodo and Kayama (1992) show that the N2 amplitude is enhanced by reaction time constaints on a go/no-go task.

One important difference in our study is that we find an N2 component where other studies have not, in the IAT task. A possible explanation for this is the stimuli used in the n-IAT task; differences exist when comparing waveforms as a function of content used in the task, i.e., pictures vs. words (Williams and Themanson, 2011). Fleischhauer et al. (2014) do not find evidence of significant effects in the N2 (as expected). Although the authors here are considering implicit measures of neuroticism, the stimuli and experimental structure are similar to ours.

Caution is warranted in interpreting the P3-like activity identified in our study as it differs from the more classical and response-locked P3b found in other IAT-EEG studies. The P3 has been shown to index attention toward self-referent materials in an IAT (Chen et al., 2014) so our discovery of the involvement of this component in an IAT responding to both congruent and incongruent self-referent mappings was somewhat expected. The P3b component (the more common P3 variant identified in existing IAT studies) is notably comprised of lower-frequency EEG activity (0–4 Hz). As we use high-pass filtering, P3-related ERP activity is highly attenuated in our ERP measures. Also, the timecourse of this P3-like activity in our study overlaps with time regions where other studies have found N400 to be present. Importantly, the N400 is not necessarily characterized by a negative deflection in the ERP waveform, as it is measured relatively as a more negative going signal with respect to other experimental conditions. The P3a is a frontal-central tending ERP and is seen in target detection tasks to novel and infrequent stimuli, and it also reflects attention mechanisms during task processing (Polich, 2007). Wavelet analysis indicates this component is partly comprised of theta band (4-8 Hz) activity (Demiralp et al., 2001a) and source localization analysis reveals a wide range of cortical generators. Our preprocessing strategy—using high-pass filtering to avoid the use of pre-stimulus baselining confounds—compromises a robust interpretation of this frontal P3-like activity with respect to existing ERP literature. Although we find, however, in a variety of alternatively explored filtering strategies this earlier occurring frontal P3-like activity remains present particularly in conjunction with its posterior counterpart (P3b). As other IAT-ERP indices have been implicated in this time-region we choose to retain this time-region in our analysis. There is strong suggestion of overlapping cortical generators of IAT-sensitive ERPs in both our N2 and P3 analysis time windows as can be seen in our LORETA analysis particularly for the incongruent block conditions. This would indicate ERP activity might not only be modulated by differences in amplitude of underlying components but also latency. We do find, however, significant patterns of correlated activity using LORETA of brain-regions typically implicated in the generation of the P3 (notably cingulate cortex and medial frontal gyrus; Volpe et al., 2007). Egenolf et al. (2013) similarly examine covarying relationships of brain-region activation to the magnitude of behavioral IAT effect and find differences in a time window of 510–710 ms between incongruent and congruent ERP activity that is proportional to the IAT effect, a time region largely corresponding to the P3 ERP.

Given previous results of the n-IAT we would expect averaged D-score across participants to be more positive (i.e., groups of participants in previous studies tended to show a stereotypic pro-nature IAT effect like we similarly have). These results might indicate the nIAT-effect cannot be measured reliably on all participants, due to differences such as motivation during the task and/or different patterns of top-down control employed in the task. The presence of such an effect is important in understanding instances where the IAT might be failing to measure an expected bias and would be a potential source of detrimental noise in measuring relationships to explicit measures.

## 5. Conclusions

The results presented in this paper indicate that EEG is informative in understanding cognitive processes behind the n-IAT. Our results both confirm patterns of activity seen in other IAT studies and also extend these by showing novel behavioral-ERP predictive relationships. Importantly, we identify that N2-/MFN- related amplitudes in our ERP analysis time window show a correlational relationship with D-score, highlighting the potential involvement of participant motivation via proactive cognitive control and top-down attention related mechanisms as a source of noise in the successful measurement of an IAT-effect. This has broad implications for other studies utilizing the n-IAT (and other IATs in general) in that it might offer an explanation as to why IAT measures can often fail to correlate with explicit measures. Such a line of evidence would indicate other secondary measures (including EEG) alongside the IAT may be useful in measuring these motivational related factors so as to enable an experimenter to disqualify participants who may be IAT averse.

One notable difference between this study and other studies is how the data are preprocessed due to the presence of pre-stimulus locked ERP activity related to the CNV. Although this potential problem of the IAT-ERP related to confounds introduced by standard baselining exists, there is little reported in the IAT literature that it has been at least taken account of.

One area of future work to be explored next is to examine predictive relationships and functional/structural brain differences that emerge within, and across, participants for a variety of IAT tasks.

## Author contributions

GH ideated the project, created the data acquisition protocol, led the data acquisition, led the data analysis, wrote the initial version of the paper and agrees to be accountable for all aspects of the work. LB contributed to the design of the work and interpretation of data for the work, revised the work for intellectual content, contributed to final approval of the version to be submitted and agrees to be accountable for all aspects of the work. AS ideated, planned and oversaw the project, reviewed and refined the paper, and agrees to be accountable for all aspects of the work.

## Funding

The work reported here was funded by the International Energy Research Centre/Enterprise Ireland and by Science Foundation Ireland under grant SFI/12/RC/2289.

### Conflict of interest statement

The authors declare that the research was conducted in the absence of any commercial or financial relationships that could be construed as a potential conflict of interest.
