# Supplementary material for: Neural Patterns of the Implicit Association Test
Source: Front Hum Neurosci. 2015 Nov 24;9:605. doi: 10.3389/fnhum.2015.00605 (PMC4656831; doi:10.3389/fnhum.2015.00605)
Supplement: Supplementary file 1 [file Presentation1.PDF]

Frontiers in Human Neuroscience

Research Article
2015November 16, 2015
123456789

Supplementary Material: Neural Patterns of the Implicit Association Test

Graham F. Healy [1,*], Lorraine Boran [2], Alan F. Smeaton [1]

[1] *Insight Centre for Data Analytics, Dublin City University, Dublin, Ireland*
[2] *School of Nursing & Human Sciences, Dublin City University, Dublin, Ireland*

Correspondence*:
Graham Healy
Insight Centre for Data Analytics, Dublin City University, Glasnevin, Dublin 9, Ireland, graham.healy@dcu.ie

1 SUPPLEMENTARY MATERIALS

BASELINING FIGURES

The issue surrounding potential confounds arising from the use of standard baselining processing in the IAT paradigm can be seen in Figure 12. Here we show a statistical map (using related t-test) showing statistical differences between pre-stimulus amplitudes in the preplanned baseline time period (i.e. -200 ms to 0 ms). There is strong suggestion of differences in ERP amplitude in this time region between conditions.

Similarly, examining reference channels (TP9 and TP10), we find using a repeated-measures ANOVA that significant differences exist between conditions at the p=.1 level: $[F(4.021, 19) = 15.586, \eta^2 = 0.136, p = 0.1]$.

DATA FOR QUADRATIC RELATIONSHIPS

Although not fully investigated, we present data for indicating evidence of quadratic relationships present in the IAT-EEG in Table 3 and Figure 3. Such findings have been highlighted by other authors.

ADDITIONAL TABLES AND FIGURES

Tables and Figures are extended to include additional plots and plots for non-significant time-windows (N1,P2) as identified by the RM-ANOVA.

2 SUPPLEMENTARY TABLES AND FIGURES

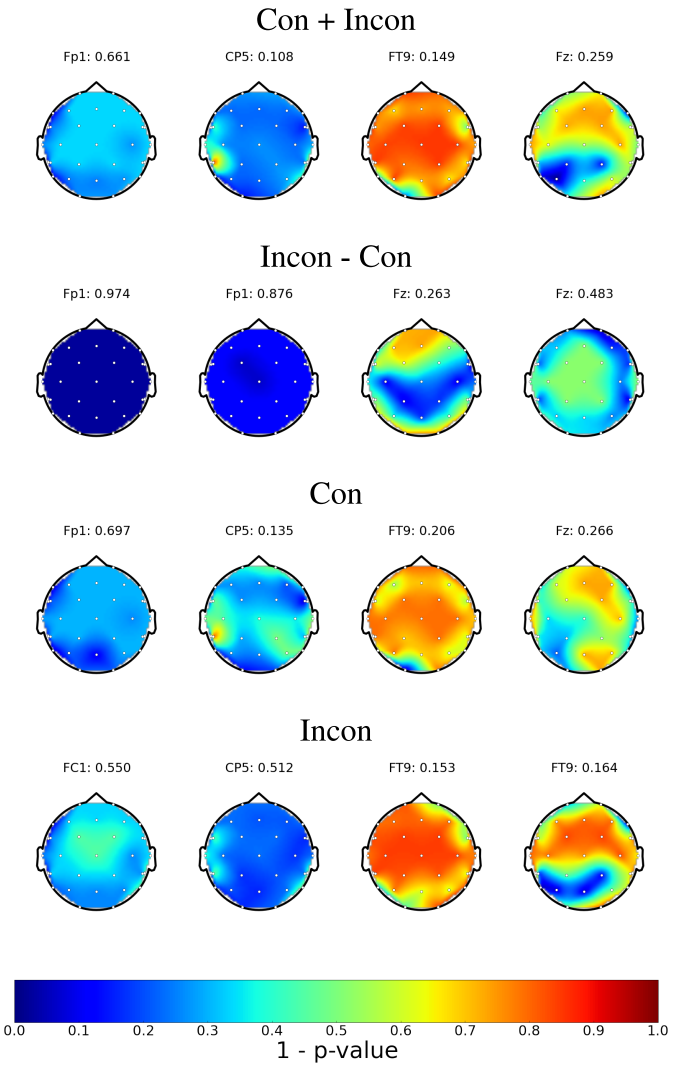

Figure 1: Scalp plots of (one-way ANOVA) p-values showing the statistical significance of differences present at electrode sites between high, medium and low d-scores. Individual plots left to right map to: N1, P2, N2 and P3. Top to bottom: (a) statistical differences between d-score groups in mean ERP amplitudes collapsed across congruent and incongruent conditions, (b) statistical differences between d-score groups in mean ERP amplitude differences between congruent and incongruent blocks, (c) statistical differences between d-score groups in mean ERP amplitude for congruent conditions, (d) statistical differences between d-score groups in mean ERP amplitude for congruent conditions. Red coloured regions indicate a lower - more statistically significant - p-value while blue indicates a higher p values.

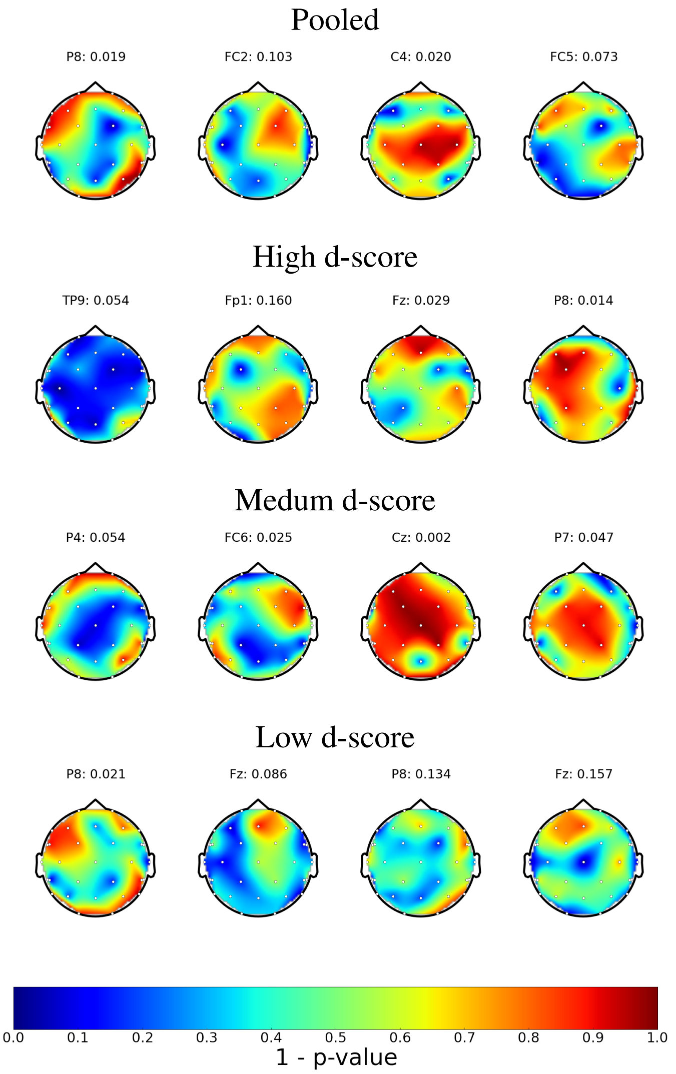

Figure 2: Scalp plots of (related t-test) p-values showing the statistical significance of mean-amplitude differences between congruent and incongruent conditions at electrode sites across time - and by d-score groups (high, medium, low). Individual plots left to right map to: N1, P2, N2 and P3. Top to bottom: (1) significance of amplitude differences between congruent and incongruent conditions irrespective of d-score grouping, (2) significance of amplitude differences between congruent and incongruent conditions for participants in the high d-score group, (3) significance of amplitude differences between congruent and incongruent conditions for participants in the medium d-score group, (4) significance of amplitude differences between congruent and incongruent conditions for participants in the low d-score group

.

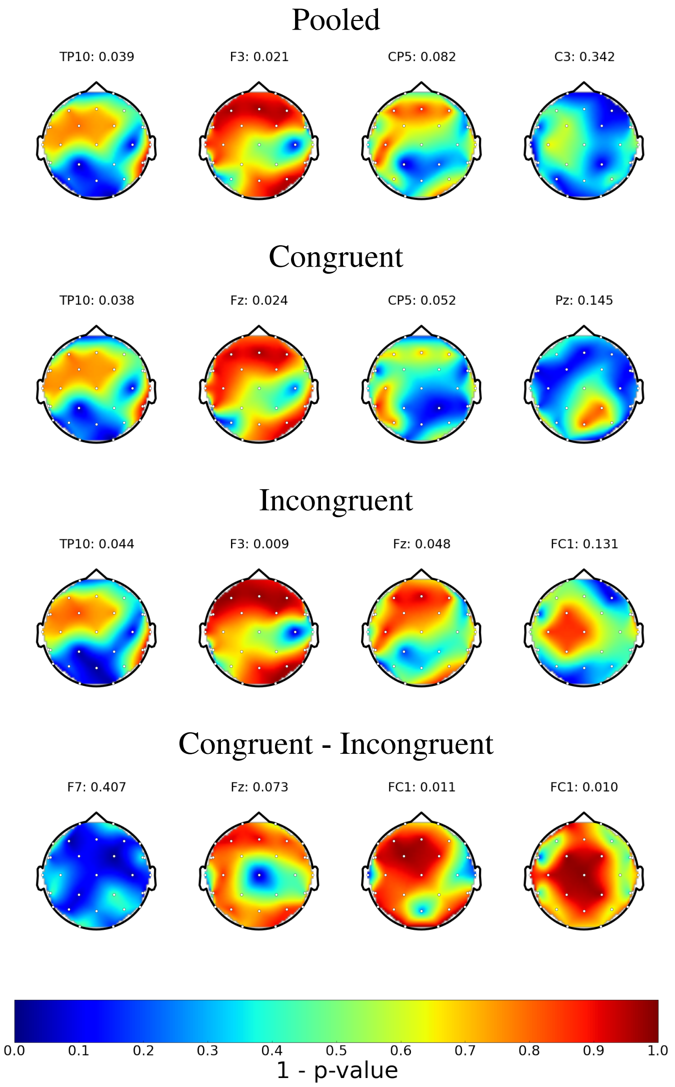

Figure 3: Scalp plots of correlational activity explained by a quadratic relationship (over a linear one) between EEG and d-score across time (via bootstrap resampling statistic examining Adjusted R^2 for linear and quadratic fittings). Individual plots left to right map to: N1, P2, N2 and P3. Top to bottom: (1) significance differences of correlational activity for pooled congruent and incongruent conditions, (2) significance differences of correlational activity for congruent conditions, (3) significance differences of correlational activity for incongruent conditions, (4) significance differences of correlational activity for difference between EEG activity for congruent and incongruent conditions.

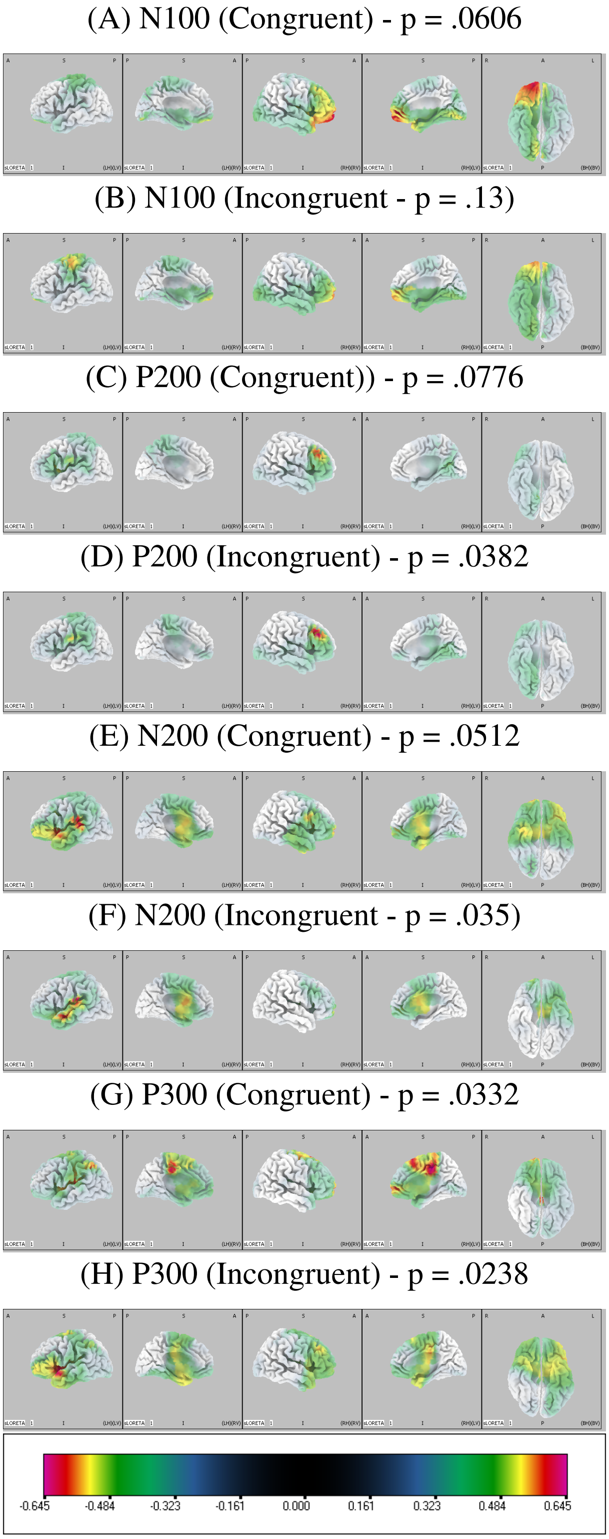

Figure 4: Correlated LORETA voxel activity and d-score. D-score is correlated with congruent and incongruent ERP time-window averages across participants localising activity driving correlated scalp EEG measures. Multiple comparison corrected p-values for peak correlations are presented on top of each condition $\times$ ERP plot.

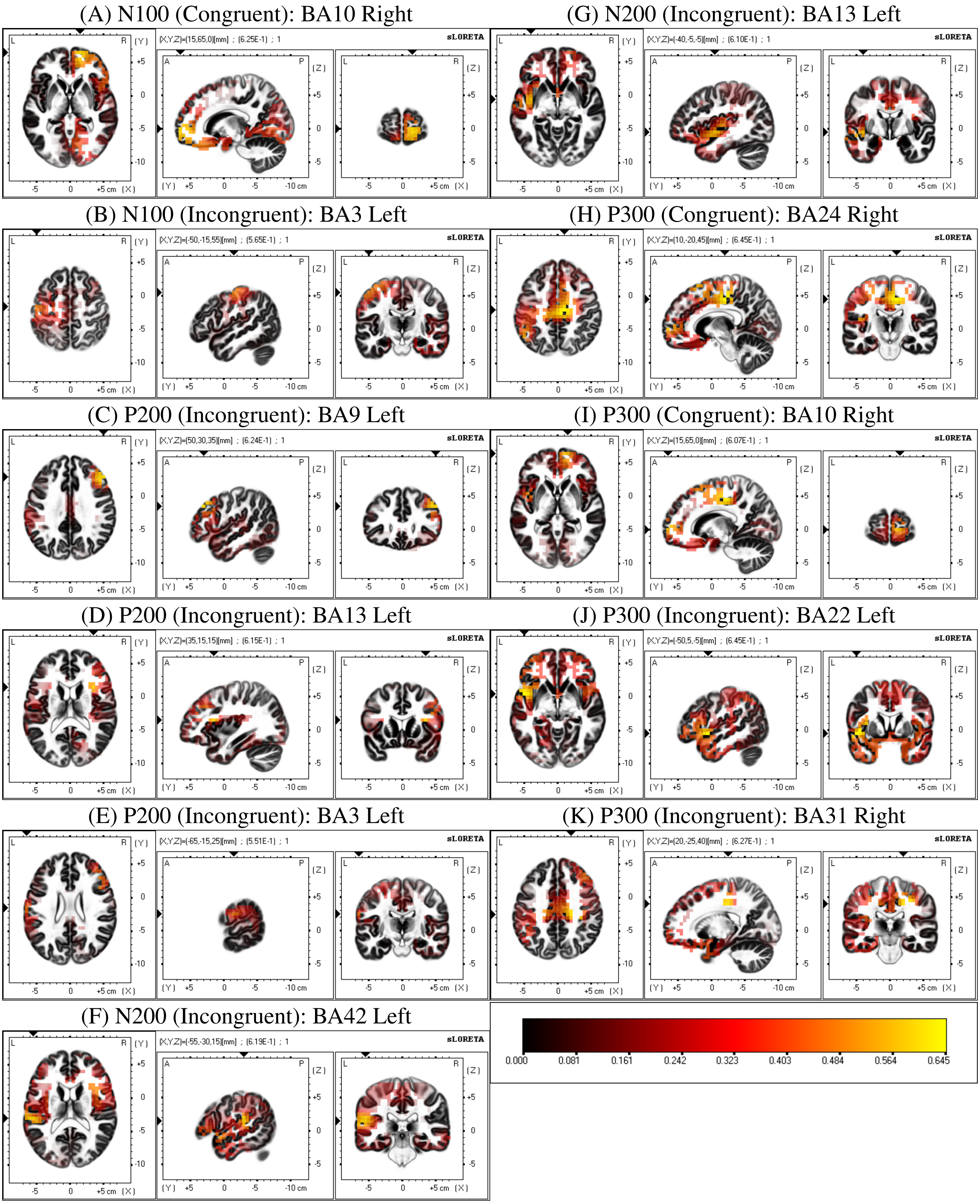

Figure 5: Selected ROIs revealed through LORETA d-score regressions.

Table 1. LORETA-derived regions of peak correlation of d-score across congruent and incongruent conditions. Rows marked with * are provided to allow comparison of matched MNI (x,y,z) coordinates between respective maximima of peak correlation between congruent and incongruent conditions.

Component	Condition	Area	Brodmann Area	Side	R	P	X	Y	Z
N100	C	Medial Frontal Gyrus*	10	R	.625	.0544	15	65	0
		Superior Frontal Gyrus	11	R	.613	.0674	15	60	10
		Insular - Sub-Lobar	13	R	.605	.0748	45	-5	15
		Postcentral Gyrus*	3	L	.432	.505	-50	-15	55
		Middle Frontal Gyrus	11	R	.578	.1152	5	65	-15
	I	Medial Frontal Gyrus*	10	R	.579	.1346	15	65	0
		Middle Frontal Gyrus	11	R	.563	.1638	5	65	-15
		Postcentral Gyrus*	3	L	.565	.1598	-50	-15	55
		Superior Frontal Gyrus*	11	R	.0	.9999	15	60	10
		Insular - Sub-Lobar*	13	R	.466	.4314	45	-5	15
P200	C	Insular - Sub-Lobar*	13	R	.589	.078	35	15	15
		Middle-Superior Frontal Gyrus	9	R	.585	.082	45	30	35
		Insular - Sub-Lobar	13	L	.539	.1538	-40	10	10
		Middle Central Gyrus*	9	R	.581	.0874	50	30	35
	I	Middle Central Gyrus	9	R	.587	.0468	50	30	35
		Insular - Sub-Lobar*	13	R	.615	.052	35	15	15
		Middle-Superior Frontal Gyrus*	9	R	.621	.048	45	30	35
		Insular - Sub-Lobar*	13	L	.391	.580	-40	10	10
N200	C	Temporal Lobe - STG	42	L	.619	.0530	-55	-30	15
		Temporal Lobe - STG	22	L	.6	.0734	-45	5	-5
		Insular - Sub-Lobar	13	L	.595	.0786	-40	5	-5
		Postcentral Gyrus*	43	L	.598	.075	-65	-20	20
		Insular - Sub-Lobar*	13	L	.550	.144	-45	0	-10
	I	Postcentral Gyrus	43	L	.643	.041	-65	-20	20
		Insular - Sub-Lobar	13	L	.605	.0812	-45	0	-10
		Temporal Lobe - STG*	42	L	.541	.190	-55	-30	15
		Temporal Lobe - STG*	22	L	.521	.230	-45	5	-5
		Insular - Sub-Lobar*	13	L	.560	.149	-40	5	-5
P300	C	Cingulate Gyrus	24	R	.645	.033	10	-20	45
		Insular - Sub-Lobar	13	L	.623	.0548	-45	-25	20
		Medial Frontal Gyrus	10	R	.605	.0732	15	60	5
		Superior Temporal Gyrus*	22	L	.534	0.1842	-50	5	-5
		Cingulate Gryus*	31	R	582	.103	20	-25	40
		Postcentral Gryus*	3	R	0	.99	30	-25	40
	I	Superior Temporal Gyrus	22	L	.645	.0244	-50	5	-5
		Cingulate Gryus	31	R	.627	.037	20	-25	40
		Postcentral Gryus	3	R	.627	.037	30	-25	40
		Cingulate Gyrus*	24	R	.570	.095	10	-20	45
		Insular - Sub-Lobar*	13	L	.501	.231	-45	-25	20
		Medial Frontal Gyrus*	10	R	.459	.333	15	60	5

Table 2. Adjusted R^2 across behavioural and EEG activity measures for linear curve fittings. Correlated variable pairs presented in the first column as *Behavioural Measure, EEG Measure*. * indicates univariate p-value $<.10$ and ** indicates univariate p-value $<.05$. Polarity of Pearson-r correlation coefficient is presented prefixed to R^2. D=d-Score, C = Congruent, I = Incongruent, rt() = Reaction Time. Max columns represent the electrode site with smallest p-value for d-score correlated with EEG measure (first 4 rows) where electrode site for each EEG measure type (C+I, C-I, C, I) is maintained across subsequent comparisons as a way to interpret the source of EEG activity driving correlations with d-score at that site.

	N100				P200			
	Fz	Cz	Pz	Max	Fz	Cz	Pz	Max
D,C-I	0.23*	0.14**	0.08	0.31*(T8)	0.00	-0.05	-0.05	-0.16**(P4)
D,C+I	-0.01	-0.04	-0.07	-0.13**(CP5)	0.06	0.14**	0.14**	0.24*(CP5)
D,I	-0.00	-0.02	-0.04	-0.12(CP5)	0.07	0.12	0.11	0.23*(CP5)
D,C	-0.02	-0.07	-0.11	-0.15**(P4)	0.07	0.17**	0.19*	0.26*(CP5)
rt(C-I),C-I	0.27*	0.17**	0.06	0.40*(T8)	0.00	-0.03	-0.03	-0.09(P4)
rt(C-I),C+I	0.01	-0.00	-0.01	-0.04(CP5)	0.00	0.04	0.11	0.10(CP5)
rt(C-I),I	0.02	0.00	-0.00	-0.03(CP5)	0.00	0.03	0.09	0.09(CP5)
rt(C-I),C	0.00	-0.01	-0.03	-0.07(P4)	0.00	0.06	0.14**	0.10(CP5)
rt(C),C-I	-0.05	-0.03	-0.03	-0.03(T8)	-0.01	-0.00	0.00	0.01(P4)
rt(C),C+I	0.02	0.04	0.13	0.07(CP5)	-0.03	-0.05	-0.09	-0.10(CP5)
rt(C),I	0.01	0.03	0.10	0.07(CP5)	-0.03	-0.05	-0.08	-0.10(CP5)
rt(C),C	0.03	0.05	0.16**	0.22*(P4)	-0.02	-0.05	-0.10	-0.10(CP5)
rt(I),C-I	0.00	0.00	-0.00	0.02(T8)	-0.01	-0.01	-0.00	-0.01(P4)
rt(I),C+I	0.04	0.03	0.09	0.02(CP5)	-0.02	-0.01	-0.02	-0.02(CP5)
rt(I),I	0.04	0.03	0.08	0.03(CP5)	-0.02	-0.01	-0.02	-0.02(CP5)
rt(I),C	0.03	0.02	0.10	0.11(P4)	-0.02	-0.01	-0.01	-0.02(CP5)

	N200				P300			
	Fz	Cz	Pz	Max	Fz	Cz	Pz	Max
D,I-C	-0.04	-0.02	-0.01	0.15**(P8)	0.12	0.16**	0.25*	0.25*(Pz)
D,C+I	-0.21*	-0.22*	-0.17**	-0.29*(C4)	0.32*	0.27*	0.12	0.39*(C4)
D,I	-0.23*	-0.24*	-0.20*	-0.29*(C4)	0.37*	0.33*	0.22*	0.42*(C4)
D,C	-0.20*	-0.20*	-0.15**	-0.29*(CP6)	0.26*	0.18**	0.02	0.30*(F4)
rt(I-C),I-C	-0.03	-0.01	-0.00	0.13**(P8)	0.09	0.12	0.14**	0.14**(Pz)
rt(I-C),C+I	-0.07	-0.10	-0.13	-0.14**(C4)	0.26*	0.21*	0.13**	0.32*(C4)
rt(I-C),I	-0.08	-0.11	-0.14**	-0.15**(C4)	0.30*	0.26*	0.19*	0.36*(C4)
rt(I-C),C	-0.06	-0.09	-0.12	-0.17**(CP6)	0.23*	0.15**	0.05	0.25*(F4)
rt(C),I-C	0.02	0.02	0.01	-0.01(P8)	-0.01	-0.01	-0.03	-0.03(Pz)
rt(C),C+I	0.13	0.13**	0.12	0.07(CP6)	-0.10	-0.11	-0.02	-0.08(F4)
rt(C),I	0.12	0.14**	0.13**	0.16**(C4)	-0.09	-0.09	-0.02	-0.07(C4)
rt(C),C	0.11	0.10	0.09	0.05(CP6)	-0.08	-0.09	-0.00	-0.06(F4)
rt(I),I-C	0.00	0.01	0.01	0.01(P8)	0.00	0.01	0.00	0.00(Pz)
rt(I),C+I	0.05	0.04	0.03	0.04(C4)	-0.00	-0.01	0.00	0.00(C4)
rt(I),I	0.04	0.04	0.03	0.04(C4)	-0.00	-0.00	0.01	0.00(C4)
rt(I),C	0.04	0.03	0.02	0.00(CP6)	-0.00	-0.01	0.01	0.00(F4)

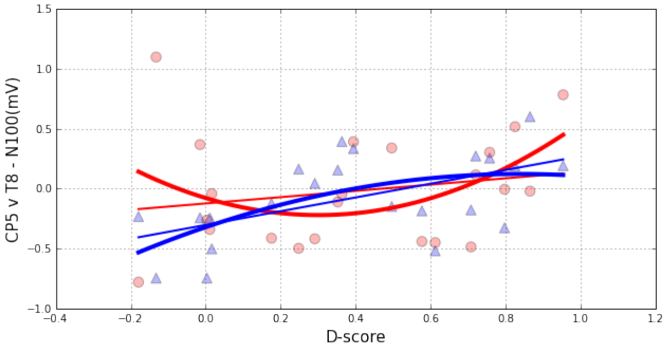

(a) N1: R_q^2=.16, p_q=.12, R_l^2=.04,p_l=.380
R_q^2=.34, p_q=.426, R_l^2=.31,p_l=.007

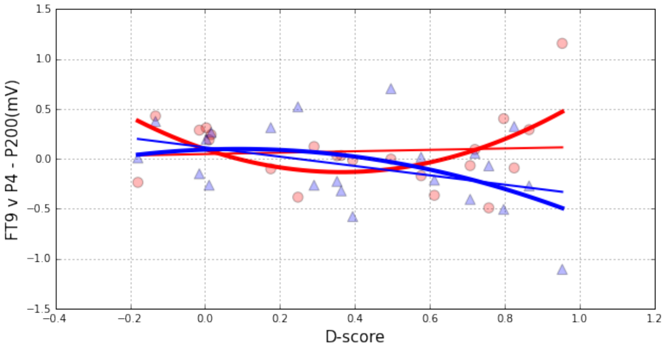

(b) P2: R_q^2=.27, p_q=.015, R_l^2=.005, p_l=.754
R_q^2=.20, p_q=.395, R_l^2=.16, p_l=.063

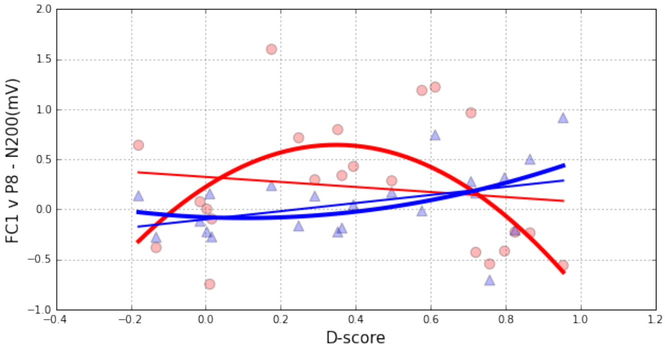

(c) N2: R_q^2=.31, p_q=.014, R_l^2=-.018,p_l=.551
R_q^2=.20, p_q=.348, R_l^2=.15, p_l=.070

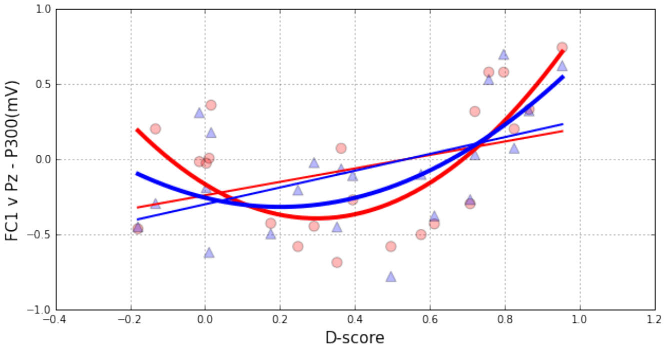

(d) P3: R_q^2=.51, p_q=.004, R_l^2=.13, p_l=.094
R_q^2=.40, p_q=.073, R_l^2=.25, p_l=.019

Figure 6: Adjusted R^2 for d-score and incongruent-congruent EEG amplitude differences for linear and quadratic curve fittings. Thick lines are for quadratic fitting and thin lines are for linear. Red lines (and first electrode site of y-axis label) are for electrode site with best quadratic fitting over a linear one (using bootstrapping). Blue lines (and second electrode site of y-axis label) are for electrode site with best linear fitting. Figure captions show adjusted R^2 for quadratic fitting, quadratic-linear difference bootstrap p-value, adjusted R^2 for linear fitting and Pearson-r p-value for linear fitting for electrode sites with best quadratic over linear fitting (first result row) and electrode sites with best linear fitting (second row).

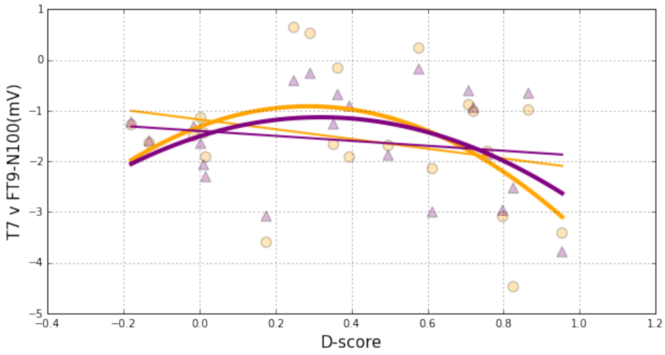

(a) N1: R_q^2=.22, p_q=.073, R_l^2=-.07,p_l=.238
R_q^2=.17, p_q=.083, R_l^2=-.03,p_l=.450

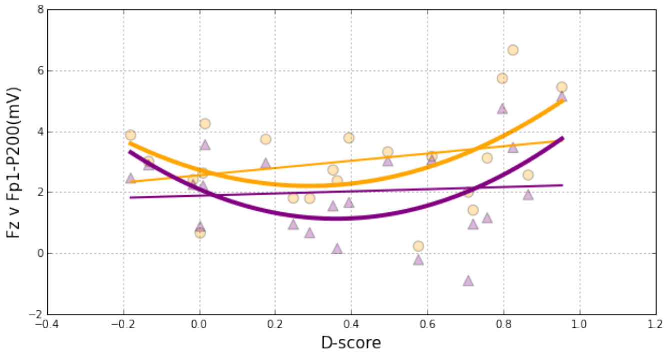

(b) P2: R_q^2=.24, p_q=.053, R_l^2=.07,p_l=.22
R_q^2=.25, p_q=.014, R_l^2=.007,p_l=.719

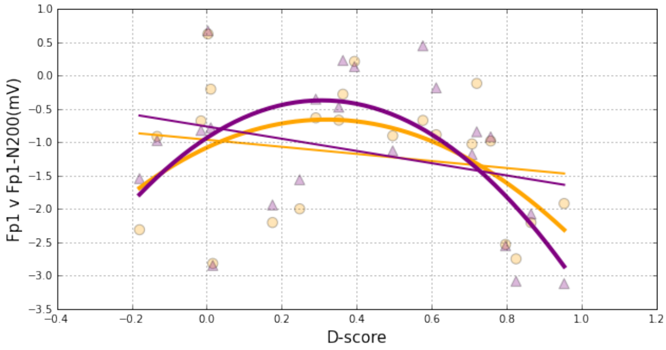

(c) N2: R_q^2=.21, p_q=.054, R_l^2=-.03,p_l=.405
R_q^2=.38, p_q=.009, R_l^2=-.08,p_l=.191

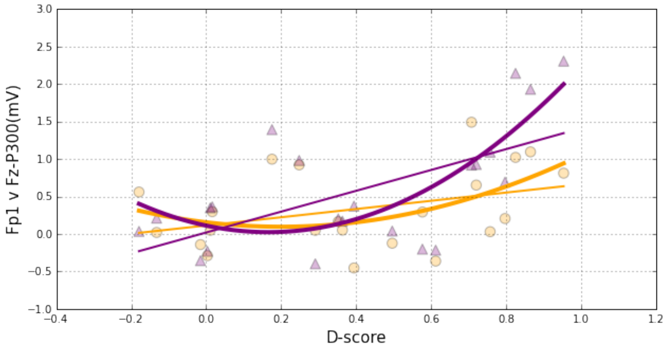

(d) P3: R_q^2=.21, p_q=.209, R_l^2=.13,p_l=.097
R_q^2=.54, p_q=.062, R_l^2=.37,p_l=.003

Figure 7: Adjusted R^2 for d-score and EEG activity measures for linear and quadratic curve fittings. Thick lines are for quadratic fitting and thin lines are for linear. Orange and purple lines are for electrode site with best quadratic fitting over a linear one (using bootstrapping) for congruent (orange) and incongruent (purple) conditions. First and second electrode site in y-axis are for selected electrode site for congruent and incongruent conditions respectively. Figure captions show adjusted R^2 for quadratic fitting, Quadratic-Linear difference bootstrap p-value, adjusted R^2 for linear fitting and Pearson-r p-value for linear fitting on the top row for congruent and the bottom row for incongruent conditions.

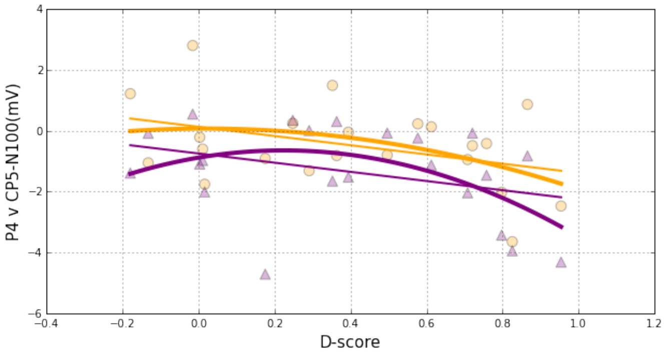

(a) N1: R_q^2=.17, p_q=.489, R_l^2=-.15,p_l=.078
R_q^2=.22, p_q=.162, R_l^2=-.12,p_l=.116

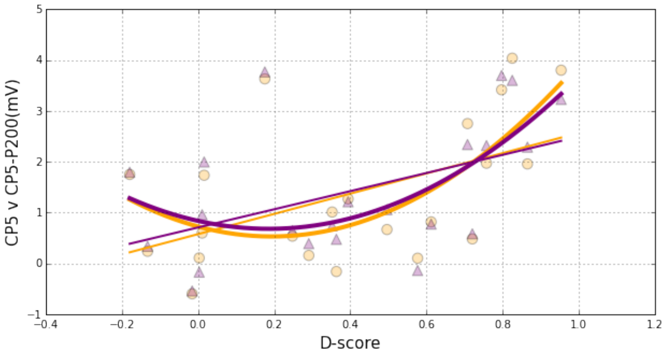

(b) P2: R_q^2=.41, p_q=.08, R_l^2=.26,p_l=.016
R_q^2=.35, p_q=.134, R_l^2=.23,p_l=.024

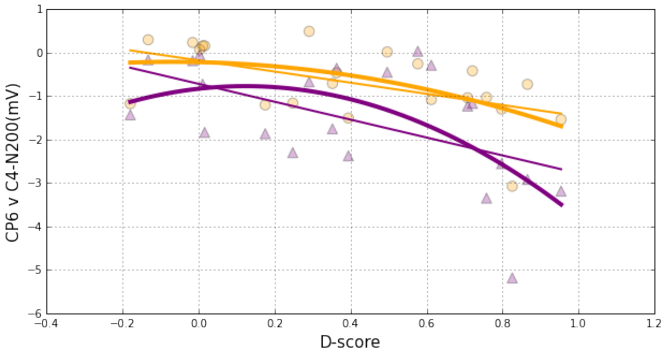

(c) N2: R_q^2=.33, p_q=.431, R_l^2=-.30,p_l=.009
R_q^2=.38, p_q=.186, R_l^2=-.29,p_l=.009

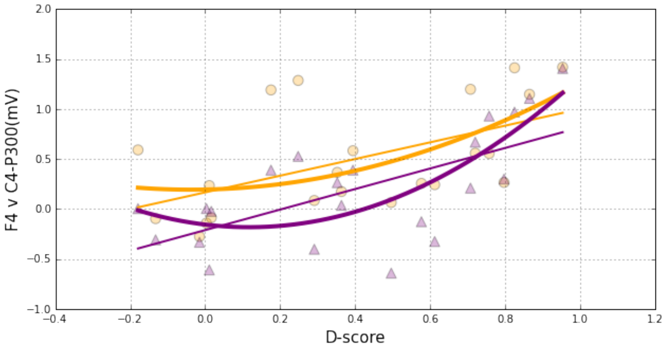

(d) P3: R_q^2=.33, p_q=.429, R_l^2=.30,p_l=.009
R_q^2=.53, p_q=.114, R_l^2=.42,p_l=.001

Figure 8: Adjusted R^2 for d-score and EEG activity measures for linear and quadratic curve fittings. Thick lines are for quadratic fitting and thin lines are for linear. Orange and purple lines are for electrode site with best linear fitting for congruent (orange) and incongruent (purple) conditions. First and second electrode site in y-axis are for selected electrode site for congruent and incongruent conditions respectively. Figure captions show adjusted R^2 for quadratic fitting, Quadratic-Linear difference bootstrap p-value, adjusted R^2 for linear fitting and Pearson-r p-value for linear fitting on the top row for congruent and the bottom row for incongruent conditions.

Table 3. Adjusted R^2 across behavioural and EEG activity measures for quadratic curve fittings. Correlatied variable pairs presented in the first column as *Behavioural Measure, EEG Measure*. ** indicates (by bootstrap comparison) the quadratic fitting had a larger adjusted R^2 over a linear fitting with a p <.10. D=D-Score, C = Congruent, I = Incongruent, rt = Reaction Time. Max columns represent electrode site with smallest p-value for d-score correlated with EEG measure (first 4 rows) where electrode site for each EEG measure type (C+I, C-I, C, I) is maintained across subsequent comparisons as a way to interpret source of EEG activity driving correlations with d-score at that site.

	N100				P200			
	Fz	Cz	Pz	Max	Fz	Cz	Pz	Max
D,C-I	0.23	0.14	0.10	0.16(CP5)	0.17	0.08	0.07	0.26**(FT9)
D,C+I	0.10	0.12	0.12	0.20(FT9)	0.26**	0.21	0.14	0.21**(Fp1)
D,I	0.09	0.09	0.08	0.17(FT9)	0.29**	0.19	0.11	0.29**(F3)
D,C	0.11	0.14	0.17	0.22(T7)	0.24	0.23	0.19	0.17(Fp1)
rt(C-I),C-I	0.29	0.18	0.06	0.02(CP5)	0.08	0.13	0.03	0.03(FT9)
rt(C-I),C+I	0.03	0.03	0.07	0.02(FT9)	0.01	0.08	0.17	0.02(Fp1)
rt(C-I),I	0.04	0.02	0.05	0.02(FT9)	0.01	0.06	0.13	0.01(F3)
rt(C-I),C	0.03	0.05	0.09	0.02(T7)	0.02	0.11	0.20	0.04(Fp1)
rt(C),C-I	0.05	0.03	0.03	0.10(CP5)	0.12	0.08	0.01	0.03(FT9)
rt(C),C+I	0.03	0.04	0.14	0.04(FT9)	0.06	0.05	0.18	0.06(Fp1)
rt(C),I	0.02	0.03	0.11	0.03(FT9)	0.08	0.06	0.15	0.07(F3)
rt(C),C	0.04	0.05	0.17	0.04(T7)	0.05	0.05	0.20	0.06(Fp1)
rt(I),C-I	0.04	0.03	0.01	0.02(CP5)	0.10	0.11	0.03	0.00(FT9)
rt(I),C+I	0.04	0.03	0.10	0.03(FT9)	0.07	0.06	0.07	0.03(Fp1)
rt(I),I	0.04	0.03	0.09	0.03(FT9)	0.05	0.04	0.05	0.06(F3)
rt(I),C	0.04	0.03	0.10	0.01(T7)	0.09	0.08	0.08	0.04(Fp1)

	N200				P300			
	Fz	Cz	Pz	Max	Fz	Cz	Pz	Max
D,C-I	0.30**	0.22**	0.06	0.31**(FC1)	0.41**	0.48**	0.40	0.51**(FC1)
D,C+I	0.35	0.28	0.18	0.30**(Fp1)	0.41	0.33	0.12	0.19(O2)
D,I	0.43	0.34	0.21	0.38**(Fp1)	0.54	0.48	0.27	0.54(Fz)
D,C	0.28	0.22	0.15	0.21(Fp1)	0.29	0.18	0.03	0.21(Fp1)
rt(C-I),C-I	0.11	0.14	0.03	0.13(FC1)	0.13	0.18	0.14	0.13(FC1)
rt(C-I),C+I	0.11	0.16	0.19	0.01(Fp1)	0.27	0.23	0.14	0.28(O2)
rt(C-I),I	0.10	0.14	0.18	0.03(Fp1)	0.30	0.26	0.20	0.30(Fz)
rt(C-I),C	0.13	0.18	0.18	0.00(Fp1)	0.25	0.19	0.05	0.19(Fp1)
rt(C),C-I	0.11	0.15	0.16	0.15(FC1)	0.07	0.11	0.16	0.09(FC1)
rt(C),C+I	0.17	0.15	0.13	0.10(Fp1)	0.10	0.11	0.06	0.12(O2)
rt(C),I	0.18	0.18	0.13	0.10(Fp1)	0.10	0.10	0.02	0.10(Fz)
rt(C),C	0.13	0.11	0.13	0.07(Fp1)	0.08	0.10	0.18	0.01(Fp1)
rt(I),C-I	0.12	0.19**	0.27**	0.15(FC1)	0.07	0.05	0.01	0.04(FC1)
rt(I),C+I	0.13	0.09	0.09	0.06(Fp1)	0.05	0.03	0.02	0.15(O2)
rt(I),I	0.09	0.06	0.05	0.02(Fp1)	0.01	0.01	0.01	0.01(Fz)
rt(I),C	0.18	0.12	0.13	0.10(Fp1)	0.09	0.06	0.04	0.05(Fp1)

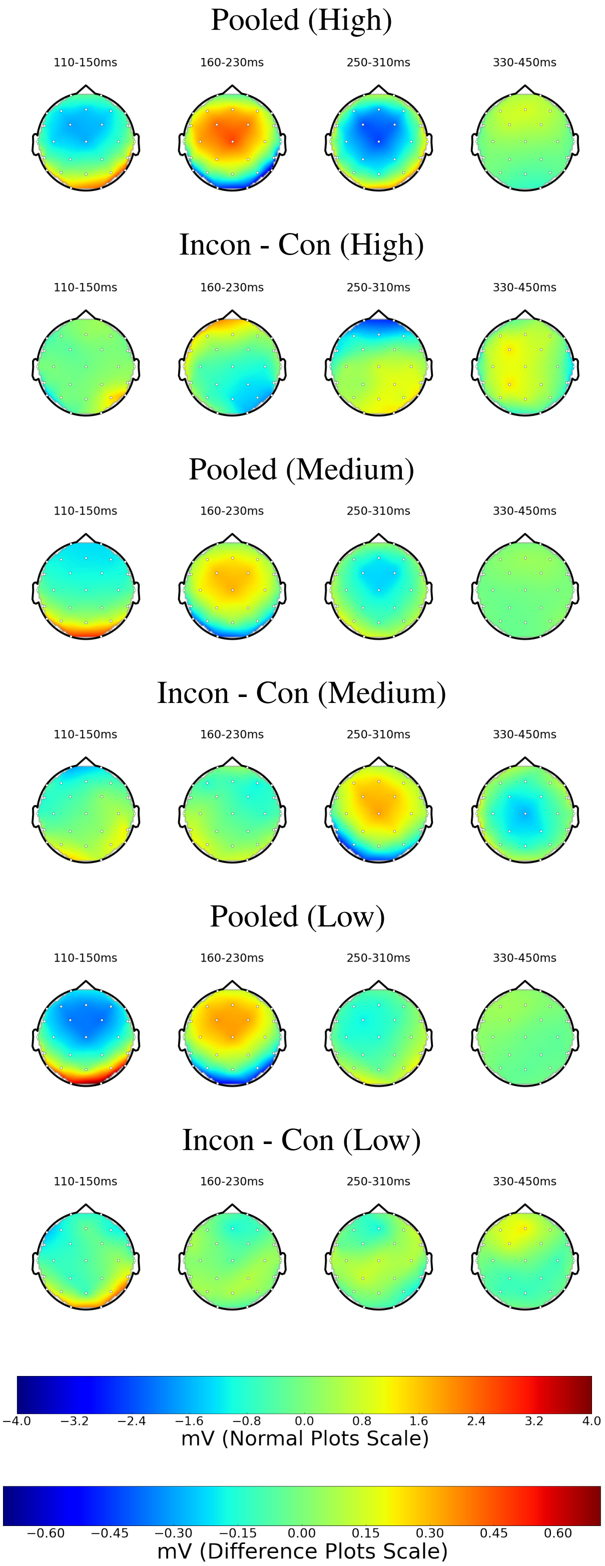

Figure 9: Scalp plots for time regions for N1, P2, N2, P3 (left to right) for high, medium and low d-scorers. Pooled and differential activity (incongruent - congruent eeg) for congruency conditions are shown for each participant.

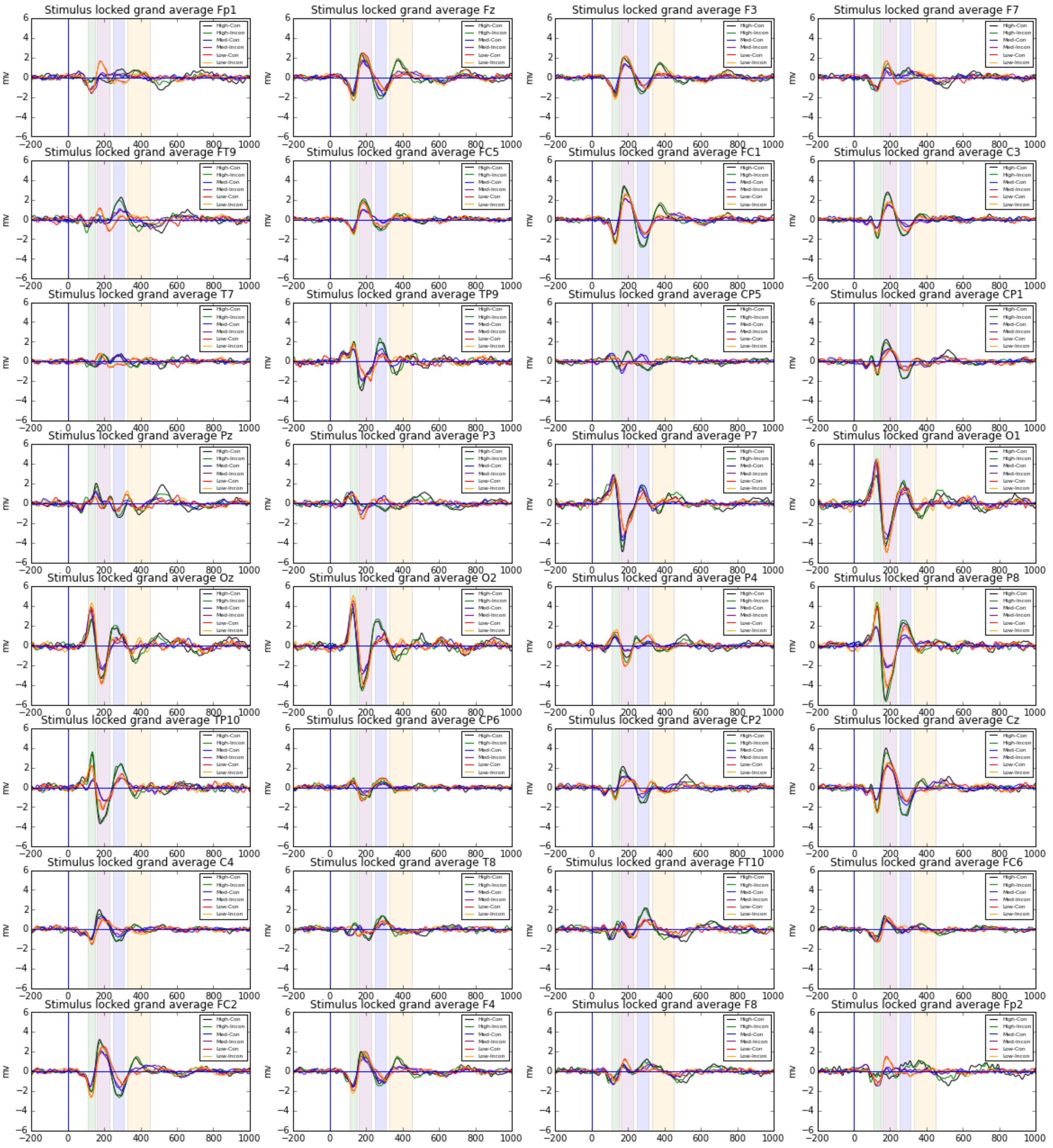

Figure 10. ERP averages across electrode sites for high, medium and low d-scorers across congruent/incongruent conditions using a CAR (common average reference). Signals are filtered in the range 4Hz-30Hz.

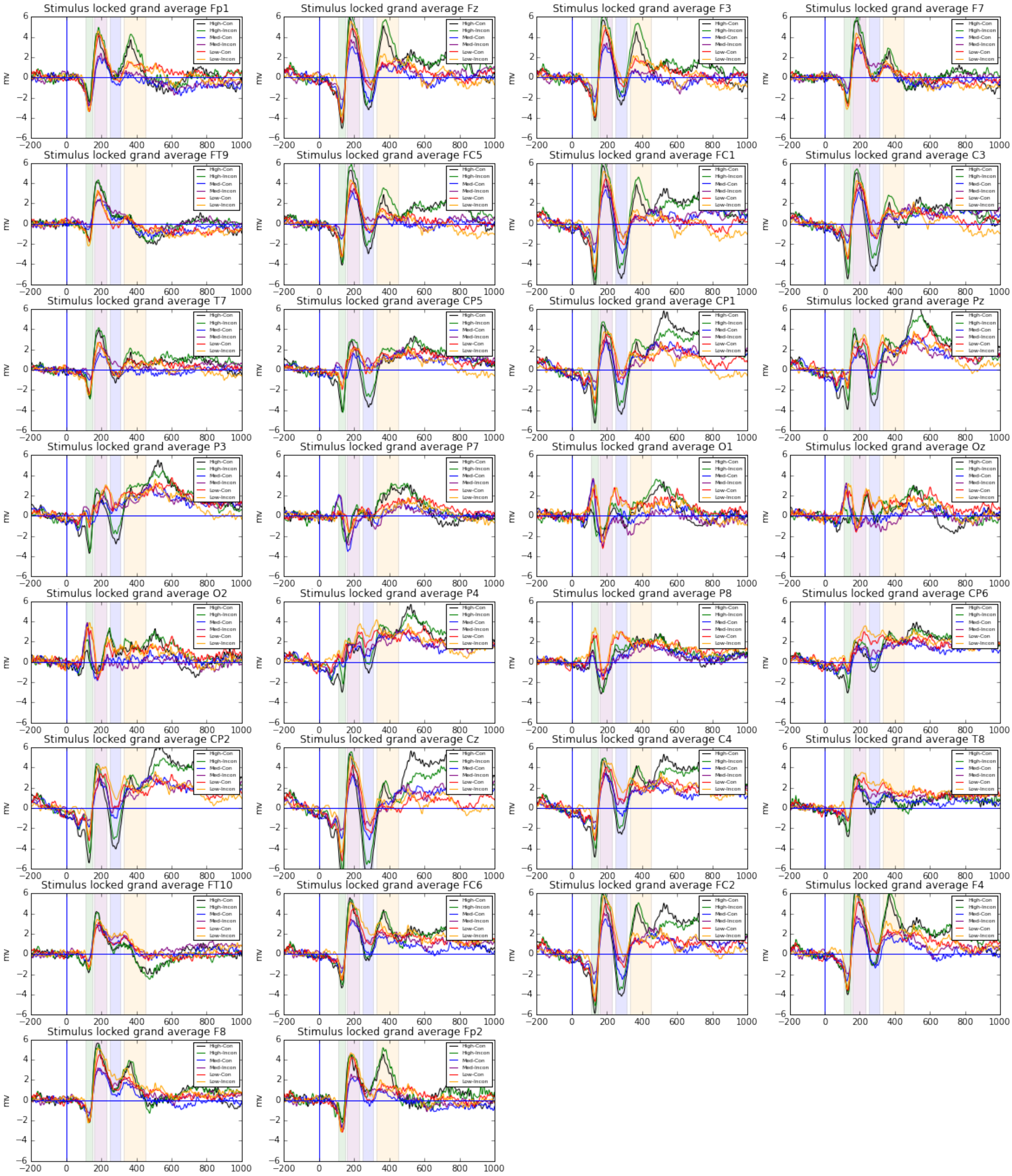

Figure 11. ERP averages across electrode sites for high, medium and low d-scorers across congruent/incongruent conditions using a TP9/TP10 reference. Signals are filtered in the range .1Hz-30Hz and no baselining procedure is applied.

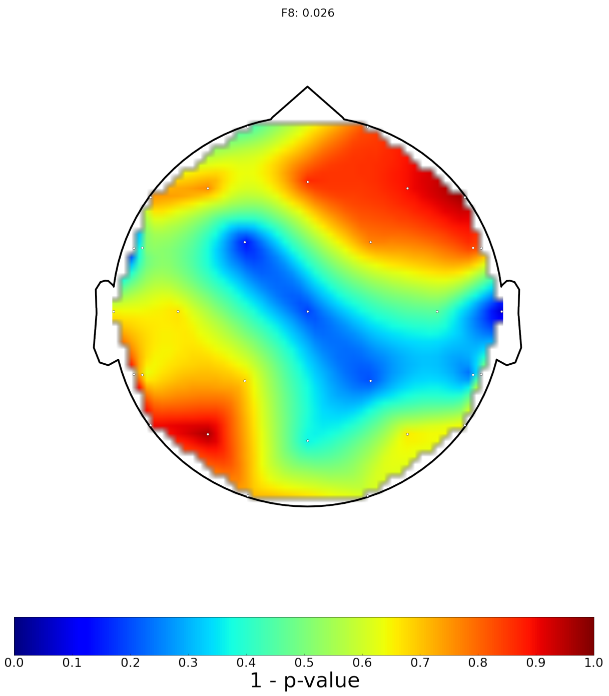

Figure 12: Scalp plot of (related t-test) p-values showing the statistical significance of mean-amplitude differences between congruent and incongruent conditions for signals filtered between .1Hz - 30Hz for the -200 ms to 0ms time range with common average reference.

.

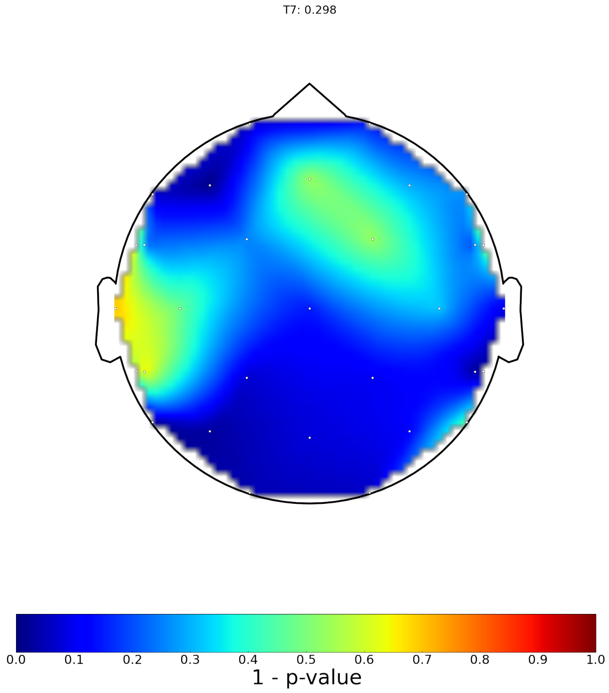

Figure 13: Scalp plot of (related t-test) p-values showing the statistical significance of mean-amplitude differences between congruent and incongruent conditions for signals filtered between 4Hz - 30Hz for the -200 ms to 0 ms time range using common average reference.

.
